# Sunlight-driven removal of tetracycline over l-histidine–MgO–AgI composites: synthesis, morphological insight, and enhanced thermal stability

**DOI:** 10.1039/d6ra08521k

**Published:** 2026-11-04

**Authors:** Bristi Dutta, Mohammad Mizanur Rahman Khan, Jae-Ho Jeong

**Affiliations:** a Department of Chemistry, Shahjalal University of Science and Technology Sylhet-3114 Bangladesh; b School of Mechanical Engineering, College of Engineering, Chung-Ang University 84 Heukseok-Ro, Dongjak-Gu Seoul 06974 Republic of Korea jaehojeong@cau.ac.kr

## Abstract

l-Histidine–MgO–AgI composites were produced by varying MgO and AgI content from 1 to 10 wt%. FTIR, UV-vis, photoluminescence (PL), and EDX data confirmed successful incorporation of MgO and AgI into l-histidine. FESEM analysis revealed a transition from relatively uniform sheet-like structures in pristine l-histidine (50–600 nm) to irregular, aggregated sheet-like morphologies in the l-histidine–MgO–AgI composites (50–800 nm). XRD analysis revealed an average crystallite size of 33.12 nm for the composites. Thermogravimetric analysis (TGA) indicated enhanced thermal stability of the composites. The composite containing 10 wt% MgO and AgI achieved the highest degradation efficiencies of 68.86% and 67.04% under sunlight and UV-lamp irradiation, respectively. HPLC analysis verified the efficient photocatalytic degradation of TC, while TOC analysis revealed a mineralization efficiency of 38.23%. The photocatalyst retained 64.19% degradation efficiency after three cycles. LC-MS analysis identified the degradation intermediates of TC. These results emphasize the potential of l-histidine–MgO–AgI composites for efficient photocatalytic removal of TC.

## Abstract 2

l-histidine–MgO–AgI composites exhibit enhanced sunlight-driven removal of tetracycline due to the synergistic effect of MgO and AgI.

## Graphical abstract

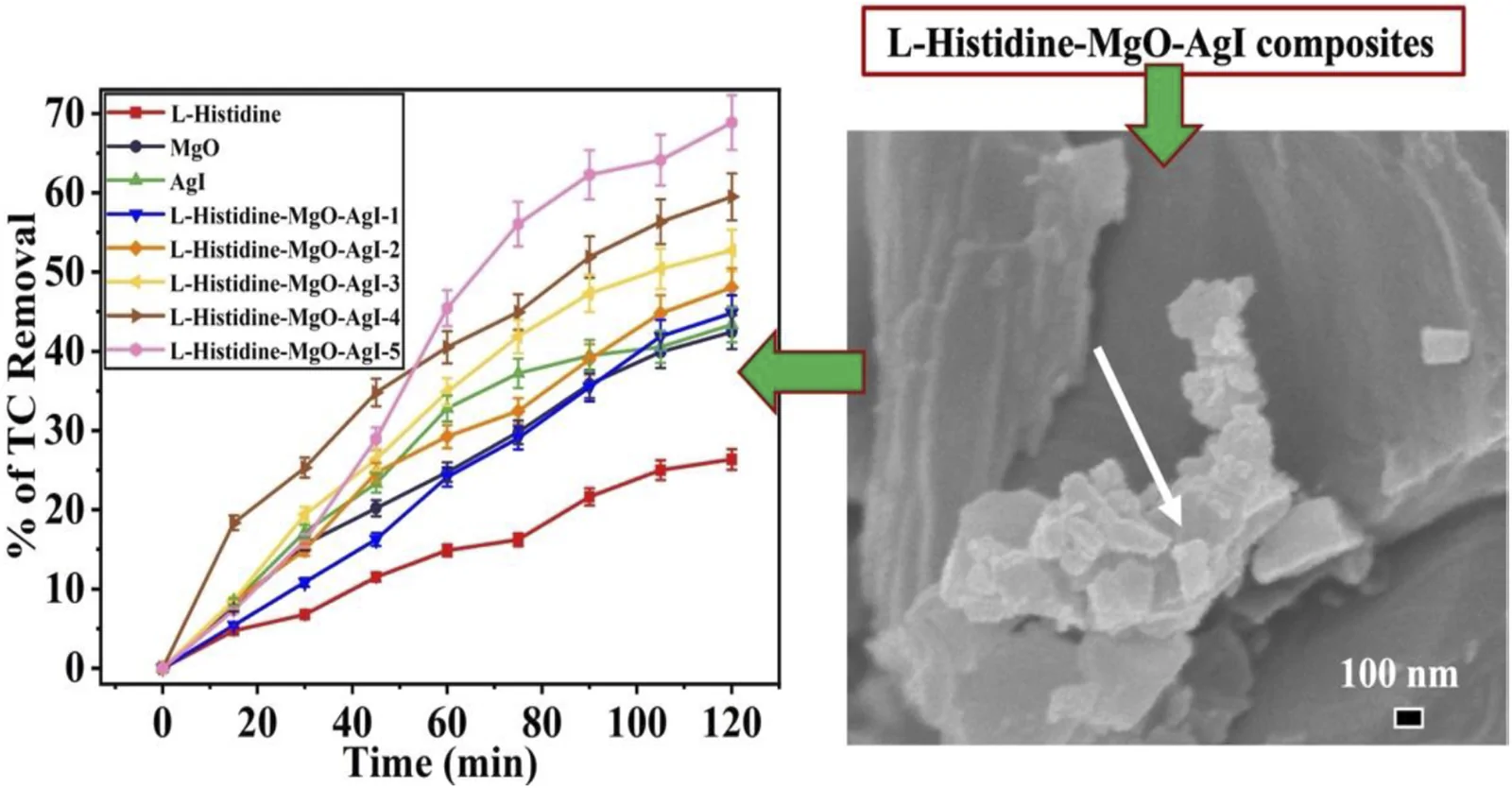

## 1. Introduction

Rapid industrial growth and expanding economic activity have intensified global concerns regarding environmental protection, ecological stability, and sustainable development. While technological advancement supports modern lifestyles and economic progress, it has simultaneously accelerated the release of harmful pollutants into natural systems, placing increasing pressure on environmental and public health.^1^ Consequently, addressing environmental degradation and developing effective remediation strategies have become scientific objectives.^2^ Among various emerging contaminants, antibiotics pose a particularly serious challenge due to their widespread application in medicine, animal husbandry, and agriculture.^3^ Continuous discharge arising from overuse and improper waste management has led to the accumulation in soil and water bodies. Such persistent exposure contributes to microbial resistance, disturbs aquatic ecosystems, facilitates bioaccumulation through food chains, and presents long-term risks to human health.^4^ Owing to their molecular stability, antibiotics are not readily decomposed by natural processes, resulting in prolonged environmental persistence.^5^ Therefore, the exploration of advanced materials capable of efficiently eliminating these pollutants has attracted considerable research attention.

Photocatalysis has emerged as one of the most promising and sustainable approaches for eliminating organic pollutants from wastewater. It offers a sustainable and low-cost solution for wastewater treatment.^6^ Various semiconductor materials have been explored as photocatalysts, among which metal oxides and metal halides have attracted considerable attention owing to their favorable electronic structures and catalytic properties.^7,8^ Magnesium oxide (MgO), a wide band gap semiconductor, possesses advantages such as chemical stability, non-toxicity, low cost, and high surface reactivity, making it a promising candidate for photocatalytic functions.^9^ However, its large band gap limits visible-light absorption and leads to rapid recombination of photogenerated charge carriers, which restricts its photocatalytic efficiency.^10^ To overcome these limitations, coupling MgO with narrow band gap semiconductors such as silver iodide has been recognized as an effective strategy. Silver iodide (AgI) exhibits strong visible-light responsiveness and excellent photosensitivity, enabling enhanced light harvesting when integrated with wide-band-gap materials.^11^ Therefore, incorporating AgI into MgO is an effective approach for photocatalytic applications. Engineering semiconductor heterojunctions has been widely recognized as an effective approach to enhance photocatalytic activity. The synergistic interaction between coupled semiconductors facilitates charge transfer across the interface, prolongs the lifetime of photogenerated charge carriers, and improves light-harvesting capability. These advantages promote the formation of reactive oxygen species and significantly enhance the degradation of organic contaminants, making heterojunction photocatalysts attractive materials for wastewater remediation.^12,13^ In recent years, amino acids have gained increasing interest as functional modifiers in semiconductor composites due to their biocompatibility and versatile functional groups.^14^ Among various amino acids, l-histidine exhibits distinctive biochemical and physiological traits that justify its application as a nutritional supplement across diverse health conditions. It features an α-amino group, a carboxylic acid functional group, and an imidazole side chain.^15^l-histidine is particularly attractive because its imidazole side chain and multiple donor sites (–NH_2_, –COOH, –NH–/–NH_3_^+^, –COO^−^) can chelate metal ions and strongly interact with inorganic surfaces.^16^ Beyond serving as a stabilizing and capping agent during synthesis, these functional groups facilitate intimate interfacial contact between the organic matrix and the MgO–AgI heterojunction. This interaction promotes uniform particle dispersion and provides abundant adsorption sites for pollutant molecules.^17^ Furthermore, the resulting organic–inorganic interface facilitates charge transfer and prolongs the lifetime of photogenerated charge carriers. This suppresses electron–hole recombination and enhances the overall photocatalytic performance.^18^ These synergistic effects contribute to enhanced luminescence properties, improved morphological features, greater thermal stability, and superior photocatalytic activity of the resulting composites. Notably, the incorporation of l-histidine into the MgO–AgI heterojunction represents a novel organic–inorganic hybrid strategy for developing efficient photocatalysts for antibiotic degradation.

To date, there are no reports on the application of l-histidine–MgO–AgI composites for the treatment of antibiotic-contaminated wastewater. Additionally, as far as we know, no comprehensive study has yet described the production of l-histidine–MgO–AgI composites using the accessible and efficient solvent casting method, and their specific performance in the photocatalytic degradation of TC has yet to be comprehensively evaluated. The present study describes the facile fabrication of l-histidine–MgO–AgI composites through a simple solvent-casting method and investigates the synergistic effect of the MgO–AgI heterojunction and l-histidine matrix on the photocatalytic degradation of TC. Specifically, the efficacy of these composites was tested through the photocatalytic degradation of TC, which served as the model contaminant. Moreover, the distinct morphological characteristics and improved thermal stability of the prepared composites compared with pristine l-histidine were also investigated.

## 2. Experimental section

### 2.1. Materials

l-Histidine (>99%, molecular weight 155.16), magnesium oxide (MgO, ≥99%), and silver iodide (AgI, ≥99%) were procured from Merck (Germany). Acetone ((CH_3_)_2_CO, ≥99.5%) was obtained from Sigma-Aldrich, while TC was purchased from the local market. All solutions were prepared using high-purity DI water. All materials, of analytical grade, were utilized as obtained without prior purification.

### 2.2. Synthesis of l-histidine–MgO–AgI composites

l-Histidine–MgO–AgI composites were synthesized through a solvent casting technique, ensuring efficient composite formation.^14^ The corresponding reaction scheme is displayed in Scheme 1. The synthesis of l-histidine–MgO–AgI composites was initiated by dissolving 0.75 g of l-histidine in 25 mL of double-distilled water. The solution was continuously stirred at room temperature for 1 h to confirm complete dissolution. Separately, predetermined amounts of MgO and AgI were each dispersed in 10 mL of distilled water. Both suspensions were magnetically stirred for 20 min, followed by ultrasonication for 1 h to achieve uniform dispersion of the particles. Individually, measured amounts of MgO and AgI were dispersed in 10 mL of distilled water, followed by magnetic stirring for 20 minutes and subsequent ultrasonication for 1 hour to ensure thorough and uniform particle dispersion. Afterwards, the MgO and AgI suspensions were gradually mixed with the l-histidine solution. The resulting mixture was then stirred continuously for 4 hours at 60 °C to facilitate solvent evaporation. The resulting composites were obtained by casting, with the final solution poured onto a clean Petri dish. The uniform composites were then placed in a desiccator and dried at room temperature for at least 48 hours. A schematic diagram depicting the fabrication steps of the l-histidine–MgO–AgI composites is presented in Fig. 1. The binary composites (l-histidine–MgO, l-histidine–AgI, and MgO–AgI) were synthesized following previously reported literature procedures.^14,19^

**Scheme 1:**
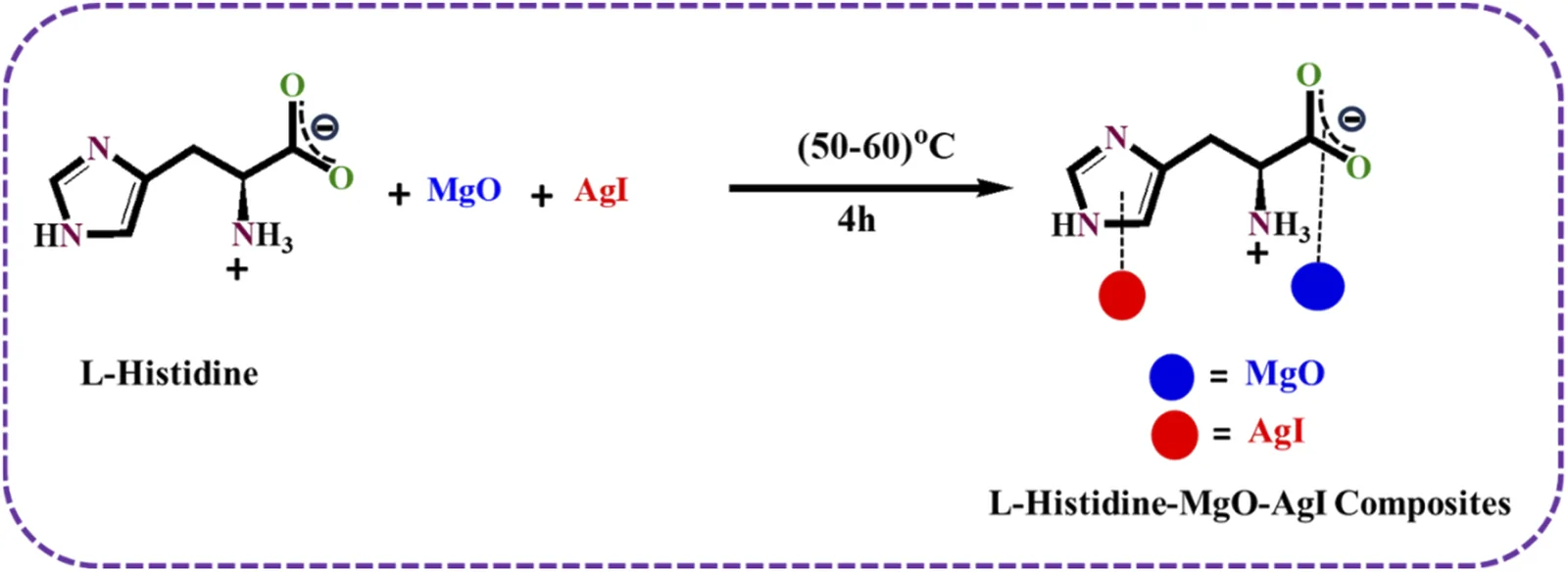
Synthetic scheme for the l-histidine–MgO–AgI composites.

**Fig. 1:**
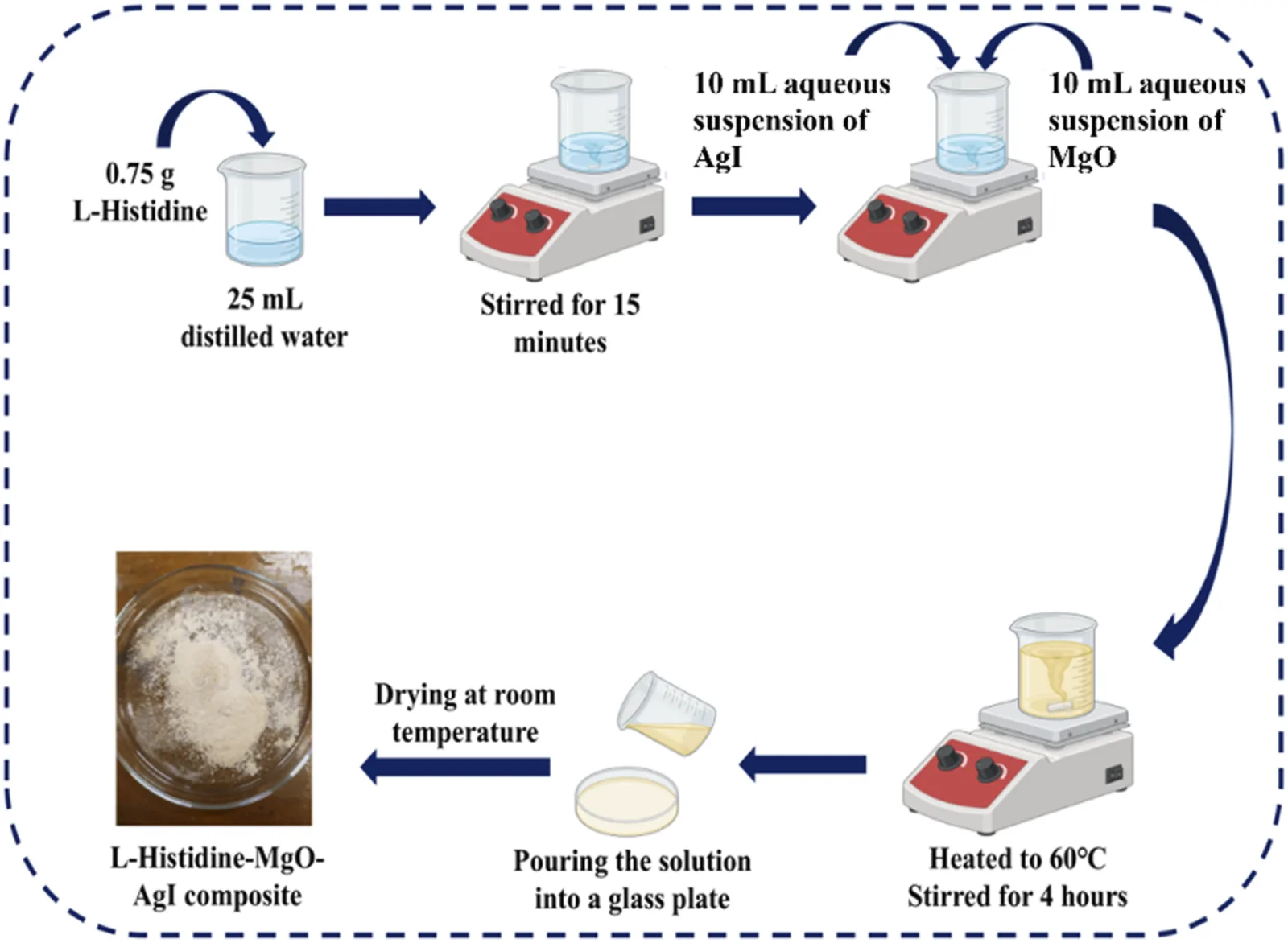
Schematic design depicting the reaction pathway for the synthesis of l-histidine–MgO–AgI composites.

By varying the MgO and AgI content from 1 to 10 wt%, five distinct batches of composites were synthesized following the same procedure. The MgO and AgI contents (wt%) are listed in Table 1, and the calculation was performed utilizing the following equation.^11^1
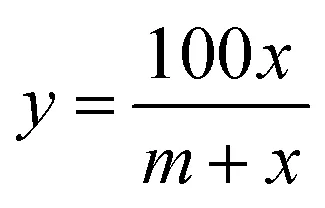
Here, *y* represents the desired weight percentage, *m* is the weight of l-histidine, and *x* denotes the amount of MgO and AgI required to achieve the target composite composition.

**Table 1:** Synthesis identifier, amount of l-histidine, MgO, and AgI utilized in the synthesis method, and type of products are indicated

Synthesis identifier	Amount of l-histidine (g)	Amount of MgO and AgI (wt%)	Type of products
l-Histidine–MgO–AgI-1	0.75	1	Composites
l-Histidine–MgO–AgI-2	0.75	3	Composites
l-Histidine–MgO–AgI-3	0.75	5	Composites
l-Histidine–MgO–AgI-4	0.75	7	Composites
l-Histidine–MgO–AgI-5	0.75	10	Composites

### 2.3. Characterization

The FTIR spectra of l-histidine, MgO, AgI, and l-histidine–MgO–AgI composites were recorded within the wavenumber range of 400–4000 cm^−1^ using a Shimadzu-IR Spirit-X series spectrometer. A Shimadzu UV-1800 spectrometer with a wavelength range of 190–800 nm was used to measure the absorbance and UV-vis spectra of l-histidine, MgO, AgI, l-histidine–MgO–AgI composites, and the antibiotic in solution. The corresponding optical band gap values were estimated utilizing Tauc plot analysis based on the UV-vis data. The PL measurements were performed at ambient temperature employing a Shimadzu RF-3501pc spectrofluorometer to evaluate the emission characteristics of the prepared samples. The morphological and compositional analyses were performed using a JEOL JSM-7600F field-emission scanning electron microscope (FESEM) equipped with an energy-dispersive X-ray (EDX) detector. Pt coating was used on the sample to measurement. X-ray diffraction (XRD) analysis was carried out using an XRD-6100 diffractometer (Shimadzu, Japan) to investigate the crystalline structure of the synthesized samples. Thermal stability was examined using TGA on a Shimadzu TGA-50H analyzer. Approximately 4.713 mg of each sample was heated from 30 °C to 850 °C at a uniform heating rate of 10 °C min^−1^ under a N_2_ environment. The concentration of TC during photocatalytic degradation was determined by high-performance liquid chromatography (HPLC) following the United States Pharmacopeia (USP, 2023) guidelines. The analysis was performed on an Agilent 1260 Infinity system equipped with Open LAB software and a Phenomenex Gemini C18 column (150 × 4.6 mm, 5 µm). A mobile phase consisting of acetonitrile and phosphate buffer (13 : 87, v/v; pH 3.0) was used at a flow rate of 1 mL min^−1^, with the column maintained at 30 °C and UV detection at 355 nm. Under these conditions, TC showed a retention time of approximately 3.9 min. The mineralization efficiency was evaluated by measuring the total organic carbon (TOC) of the treated TC solution using a TOC-VCPH analyzer (Shimadzu, Japan). The percentage TOC removal was calculated according to the method reported in the literature.^20^ The degradation products were identified by liquid chromatography-mass spectrometry (LC-MS) using an Agilent 6545 Q-TOF system equipped with an electrospray ionization (ESI) source operating in positive ion mode. Separation was achieved on a C18 reverse-phase column (2.1 × 100 mm, 1.7 µm) using a gradient elution of 0.1% formic acid in water and 0.1% formic acid in acetonitrile at a flow rate of 0.3 mL min^−1^.

### 2.4. Photocatalytic activity

To examine the catalytic efficiency, a set of photocatalytic degradation tests was performed for l-histidine, MgO, AgI, and l-histidine–MgO–AgI composites following the procedure outlined in previous studies.^14,15^ In each trial, 10 mg of catalyst was dispersed in 100 mL of a 5 mg L^−1^ TC solution within a beaker. The reaction system was then exposed to direct sunlight for 2 hours to assess the degradation efficiency. Before illumination, the mixture of catalyst and TC was stirred in the dark for 15 minutes to achieve adsorption–desorption equilibrium, following previously reported procedure.^21^ Subsequently, the suspension was irradiated under sunlight, and 3 mL aliquots were taken at certain intervals. Each withdrawn sample was centrifuged at 6000 rpm for 3 minutes to remove residual catalyst particles, and the remaining TC concentration was estimated utilizing a UV-vis spectrophotometer at its characteristic absorption wavelength of 357 nm. In addition to sunlight irradiation, photocatalytic degradation experiments were conducted under UV-lamp irradiation using the same experimental conditions. The photocatalyst's degradation efficiency was evaluated using the following formula:^14^2
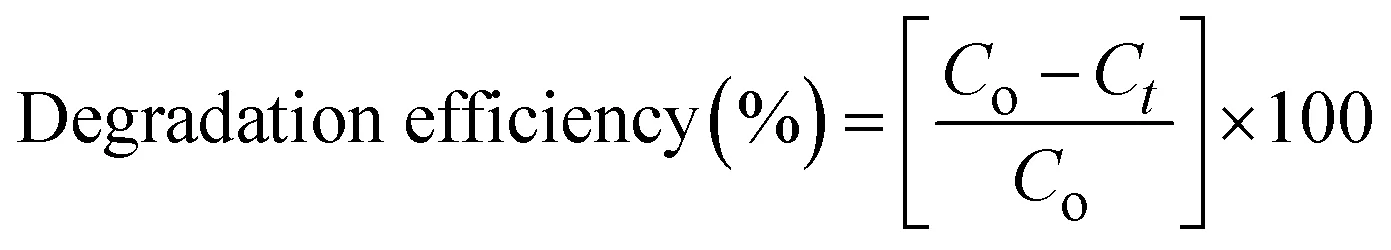
Here, *C*_o_ refers to the concentration of TC at the initial, and *C*_*t*_ indicates the TC concentration in the solution after certain periods of irradiation. Key factors, including TC concentration, catalyst amount, pH, coexisting ions, temperature, and scavenger, were systematically examined to enhance the efficiency of the photocatalytic degradation process. Control tests without the catalyst under sunlight and with the catalyst in the dark showed minimal degradation, proving that sunlight and the catalyst are both necessary for photodegradation.^22^ To validate the photocatalytic degradation of tetracycline (TC), the concentration of TC during the degradation process was determined using high-performance liquid chromatography (HPLC). For the temperature-dependent experiments, the reaction beaker was placed in a water bath heated on a hot plate equipped with a temperature probe. The bath temperature was set to 40, 50, or 60 °C and maintained under continuous stirring throughout the 240 min of sunlight irradiation. The temperature was monitored periodically using a thermometer.

#### 2.4.1. Pseudo first-order kinetics

The photocatalytic degradation kinetics were examined based on the pseudo-first-order rate law, which follows the Langmuir–Hinshelwood (L–H) model. Eqn (3) was employed to evaluate the reaction kinetics, following the approach used in previous literature for the pseudo-first-order kinetic model.^23^

3 −ln(*C*_*t*_/*C*_o_) = *kt*

Where *C*_*t*_ refers to the TC concentration at a specific time *t*, *C*_o_ indicates the initial concentration, *t* represents the exposure period, and *k* (min^−1^) denotes the reaction rate constant.^24^ The findings of this research provide a clearer perspective on the photocatalytic performance of l-histidine–MgO–AgI composites in comparison with traditional systems. Furthermore, the study underscores their potential use in environmental cleanup, especially for the breakdown of TC and other organic pollutants in water under solar irradiation. The experimental approach adopted here deepens the understanding of the functional efficiency of l-histidine–MgO–AgI composites, confirming their potential for effective photocatalytic wastewater treatment.

## 3. Results and discussion

### 3.1. FTIR spectra and analysis

FTIR analysis was conducted for l-histidine, l-histidine–MgO–AgI composites, MgO, and AgI, as shown in Fig. 2 and 3, respectively, and the corresponding peaks are compiled in Table 2.

**Fig. 2:**
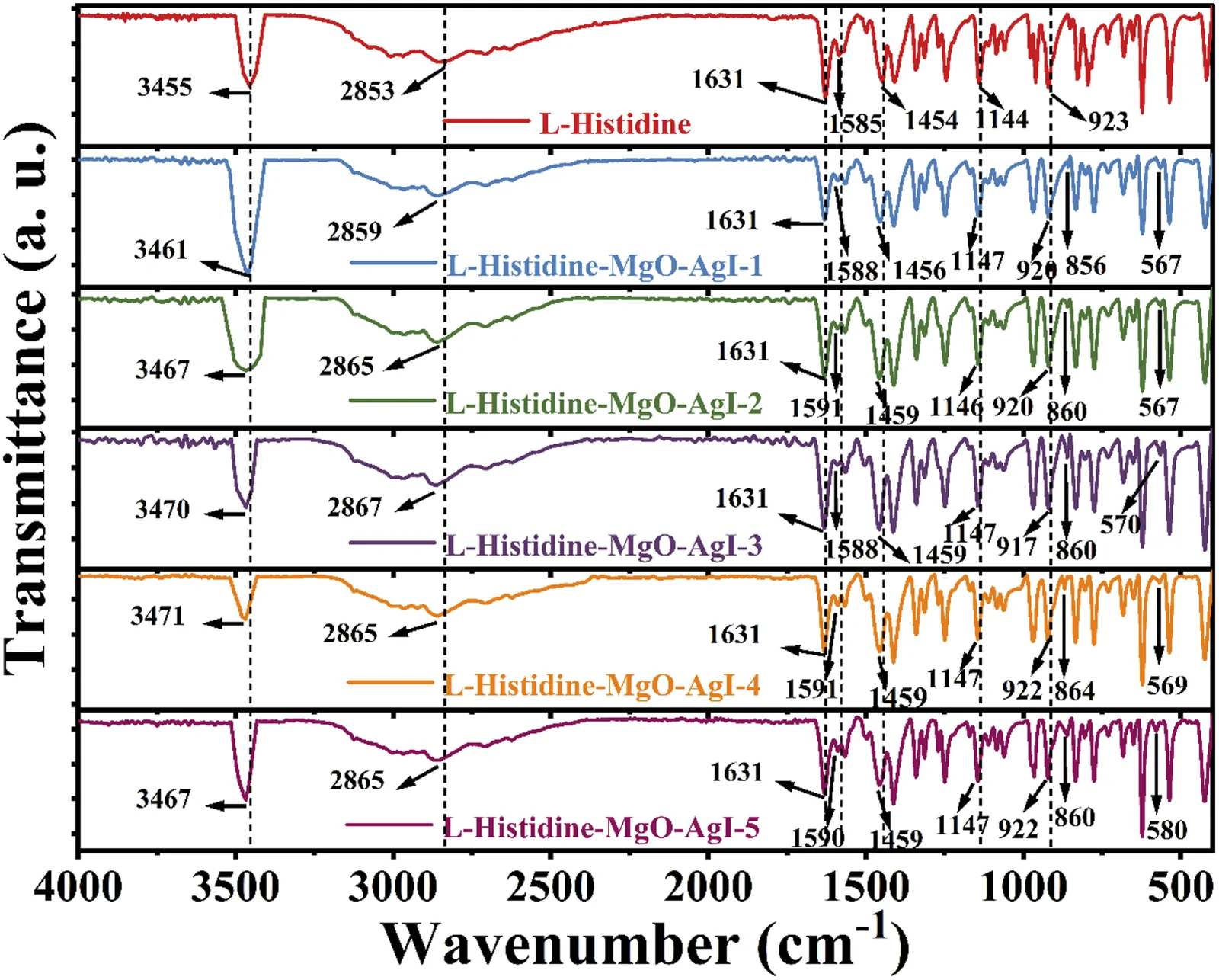
FTIR spectra of l-histidine and l-histidine–MgO–AgI composites.

**Fig. 3:**
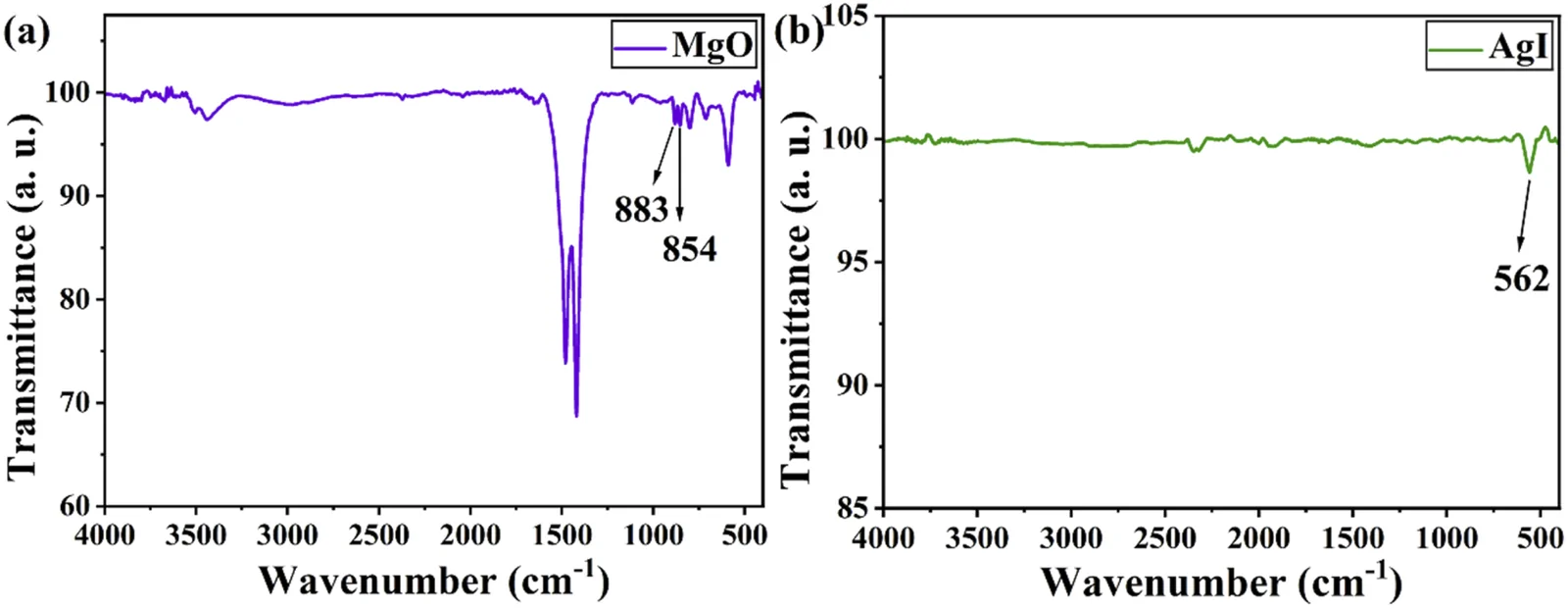
FTIR spectra of (a) MgO, and (b) AgI.

**Table 2:** Characteristic FTIR Peaks of l-histidine, MgO, AgI, and l-histidine–MgO–AgI composites

Peak assignment	Peak values of the corresponding samples (cm^−1^)					
	l-Histidine	l-Histidine–MgO–AgI-1	l-Histidine–MgO–AgI-2	l-Histidine–MgO–AgI-3	l-Histidine–MgO–AgI-4	l-Histidine–MgO–AgI-5
NH_3_^+^ stretching	3455	3461	3467	3470	3471	3467
C–H stretching	2853	2859	2865	2867	2865	2865
COO^−^ stretching	1631	1634	1635	1634	1633	1633
C=C stretching	1585	1588	1591	1588	1590	1591
N–H bending	1454	1454	1459	1459	1459	1459
C–N stretching	1144	1147	1146	1147	1147	1147
Ring stretching	923	920	920	917	922	922
Mg–O bond	—	856	860	860	864	860
Ag–I bond	—	567	567	570	569	580

l-Histidine displays several well-defined vibrational features in its FTIR spectrum, which serve as reference points for evaluating chemical interactions in the composite materials. The characteristic NH_3_^+^ stretching band appears at 3455 cm^−1^, while the aliphatic C–H stretching mode is observed at 2853 cm^−1^.^25,26^ The carboxylate (COO^−^) asymmetric stretching vibration, one of the most prominent signatures of amino acids, is located at 1631 cm^−1^.^16^ Additionally, the imidazole ring of histidine gives rise to the C=C stretching band at 1585 cm^−1^, accompanied by an N–H bending vibration at 1454 cm^−1^, C–N stretching near 1144 cm^−1^, and a distinct ring-stretching mode at 923 cm^−1^.^25,27^ In all l-histidine–MgO–AgI composite samples, these peaks remain present but exhibit slight shifts, indicating interactions between histidine functional groups and both MgO and AgI. The NH_3_^+^ and COO^−^ stretching peaks shift to the 3461–3471 cm^−1^ and 1633–1635 cm^−1^ ranges, respectively. Furthermore, smaller but noticeable shifts in the C=C, N–H, and C–N vibrational bands indicate slight modifications within the imidazole ring. A distinct Mg–O vibrational band appeared at 854–856 cm^−1^ in the FTIR spectra for every l-histidine–MgO–AgI composite sample (Fig. 2).^28^ The absorption band observed around 1476 cm^−1^ in the MgO spectrum may be attributed to carbonate species formed by the adsorption of atmospheric CO_2_ on the MgO surface.^29^ In all l-histidine–MgO–AgI composites, the FTIR spectra consistently displayed an Ag–I band at 567–580 cm^−1^ in every l-histidine–MgO–AgI composite.^30^ Thus, the FTIR analysis indicates the successful incorporation of MgO and AgI into the l-histidine. These outcomes are consistent with the previous report for the synthesis of amino acid-based composites.^15^

### 3.2. UV-visible spectra and analysis

UV-vis absorption analysis was accomplished to explore the interaction of MgO and AgI with the l-histidine as well as to estimate their band gap. The corresponding UV-vis spectra are presented. in Fig. 4.

**Fig. 4:**
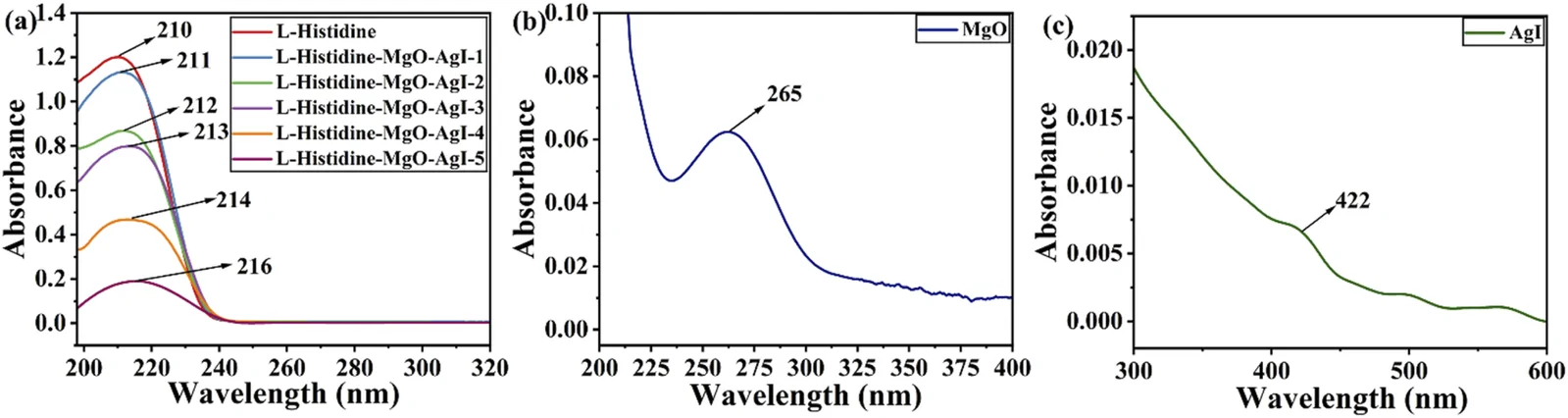
UV-vis spectra of (a) l-histidine–MgO–AgI composites, (b) MgO, (c) AgI.

In Fig. 4a, the distinct absorption peak of l-histidine appears near 210 nm and is ascribed to the π–π* transition from the C=C bond in the imidazole ring.^26^ The characteristic peak of MgO appears at 266 nm, while AgI exhibits its intrinsic absorption at 422 nm (Fig. 4b and c).^31,32^ After adjustment of the spectral positions, the composite absorption peaks were observed at 212 nm for l-histidine–MgO–AgI-1, 213 nm for l-histidine–MgO–AgI −2, 214 nm for l-histidine–MgO–AgI −3, 215 nm for l-histidine–MgO–AgI-4, and 216 nm for l-histidine–MgO–AgI-5 (Fig. 4a). The slight red shift from 210 nm to higher wavelengths in all composite samples can be ascribed to the formation of coordinated interactions between l-histidine and the MgO/AgI particles.^26^ This spectral displacement confirms the effective incorporation of MgO and AgI within l-histidine. Therefore, the UV-visible results verify the effective interaction and composite formation among l-histidine, MgO, and AgI.

The band gap energy of a composite is the minimum energy needed to move an electron from the valence band to the conduction band. Determining this energy accurately is critical for understanding the material's photophysical and photochemical behaviors. This value is particularly important for evaluating the photocatalytic performance of semiconductors, as it governs their ability to absorb light and initiate photocatalytic reactions.^33^ The band gap energies for l-histidine, MgO, AgI, and l-histidine–MgO–AgI composites were determined by applying the Tauc plot approach, which calculates the band gap by extending the straight-line segment of a graph plotting (*αhν*)^2^*versus* photon energy (*hν*) to intersect the energy axis.^14^ The Tauc approach assumes that the absorption coefficient, which varies with photon energy, can be represented mathematically by an equation relating the product of the absorption coefficient and photon energy to the difference between photon energy and the band gap energy. This relationship forms the basis for determining the band gap from optical absorption data. The equation is typically given as:

4 (*αhν*)^*n*^ = *β*(*hν* − *E*_g_)

Planck's constant is represented by *h*; *ν* denotes the photon frequency, *E*_g_ represents the band gap energy, and *β* is a constant. The parameter *n* differs depending on the sort of electronic transition, taking a value of 1/2 for indirect transitions and 2 for direct transitions.^34^ Using the Tauc plot, band gap estimation of l-histidine, MgO, AgI, l-histidine–MgO–AgI-1, l-histidine–MgO–AgI-2, l-histidine–MgO–AgI-3, l-histidine–MgO–AgI-4, l-histidine–MgO–AgI-5 is displayed in Fig. 5(a–h).

**Fig. 5:**
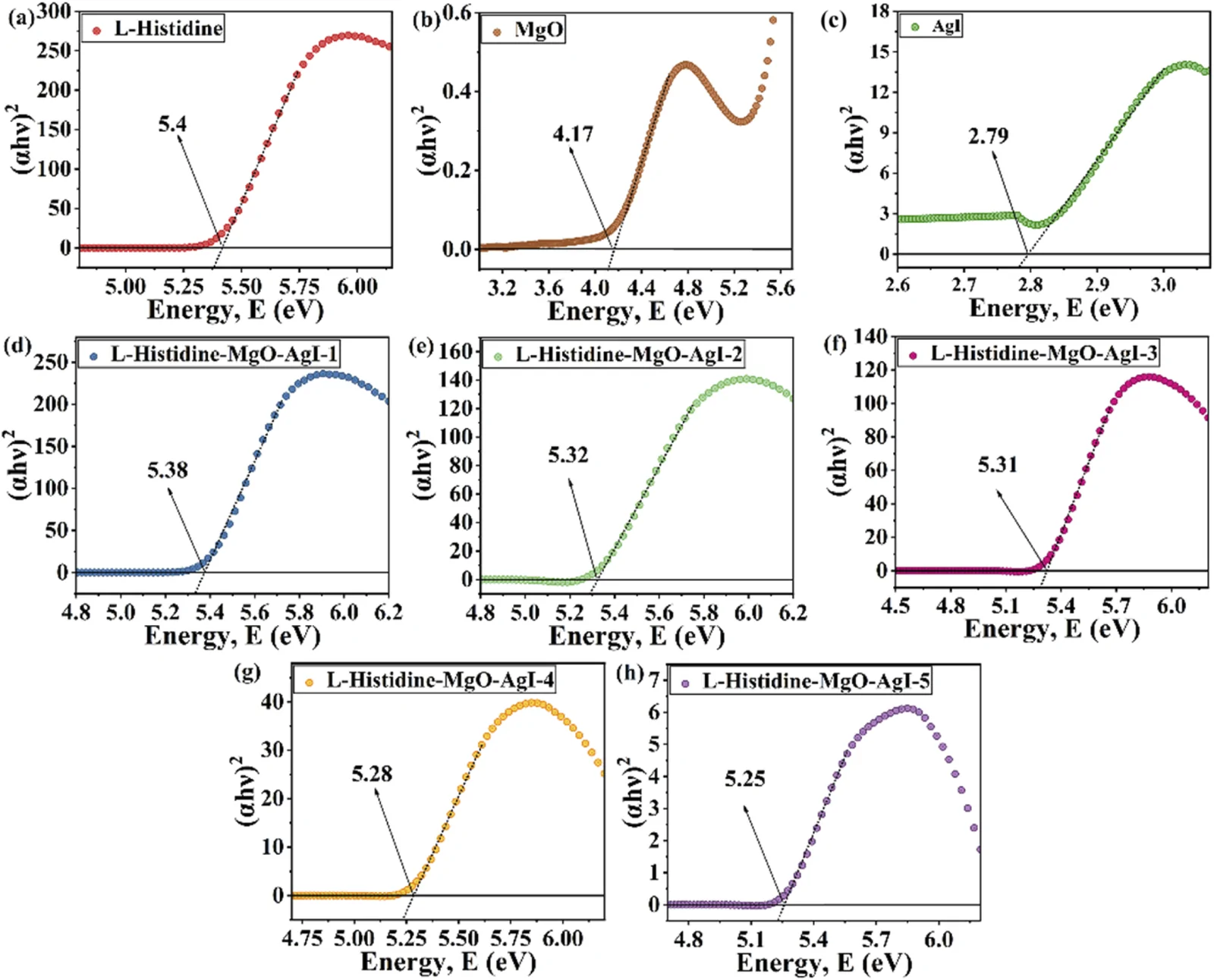
Band gap estimation of (a) l-histidine, (b) MgO, (c) AgI, (d) l-histidine–MgO–AgI-1, (e) l-histidine–MgO–AgI-2, (f) l-histidine–MgO–AgI-3, (g) l-histidine–MgO–AgI-4, (h) l-histidine–MgO–AgI-5 using the Tauc plot.

The band gap of l-histidine was calculated to be 5.4 eV, aligning with its characteristic large band gap of organic material properties.^27^ For MgO, the measured band gap was at 4.17 eV.^35^ AgI exhibited a band gap of 2.79 eV.^36^ The l-histidine–MgO–AgI composites showed varying optical behaviors, with band gaps of 5.38 eV, 5.32 eV, 5.31 eV, 5.28 eV, and 5.25 eV for samples l-histidine–MgO–AgI-1 through l-histidine–MgO–AgI-5, respectively. Incorporation of MgO and AgI within the l-histidine matrix resulted in a decrease in the composite's energy gap, which is shown in Fig. 5. These differences in values of band gap across the composites likely arise from variations in their morphology, composition, or interfacial interactions among l-histidine, MgO, and AgI components. Overall, this study demonstrates that forming l-histidine–MgO–AgI composites allows for successful tuning of the band gap, highlighting their potential utility in photocatalytic applications.

### 3.3. Photoluminescence spectra (PL) and analysis

The PL investigation was carried out to verify the optical interaction among l-histidine, MgO, and AgI in the synthesized l-histidine–MgO–AgI composites (Fig. 6).

**Fig. 6:**
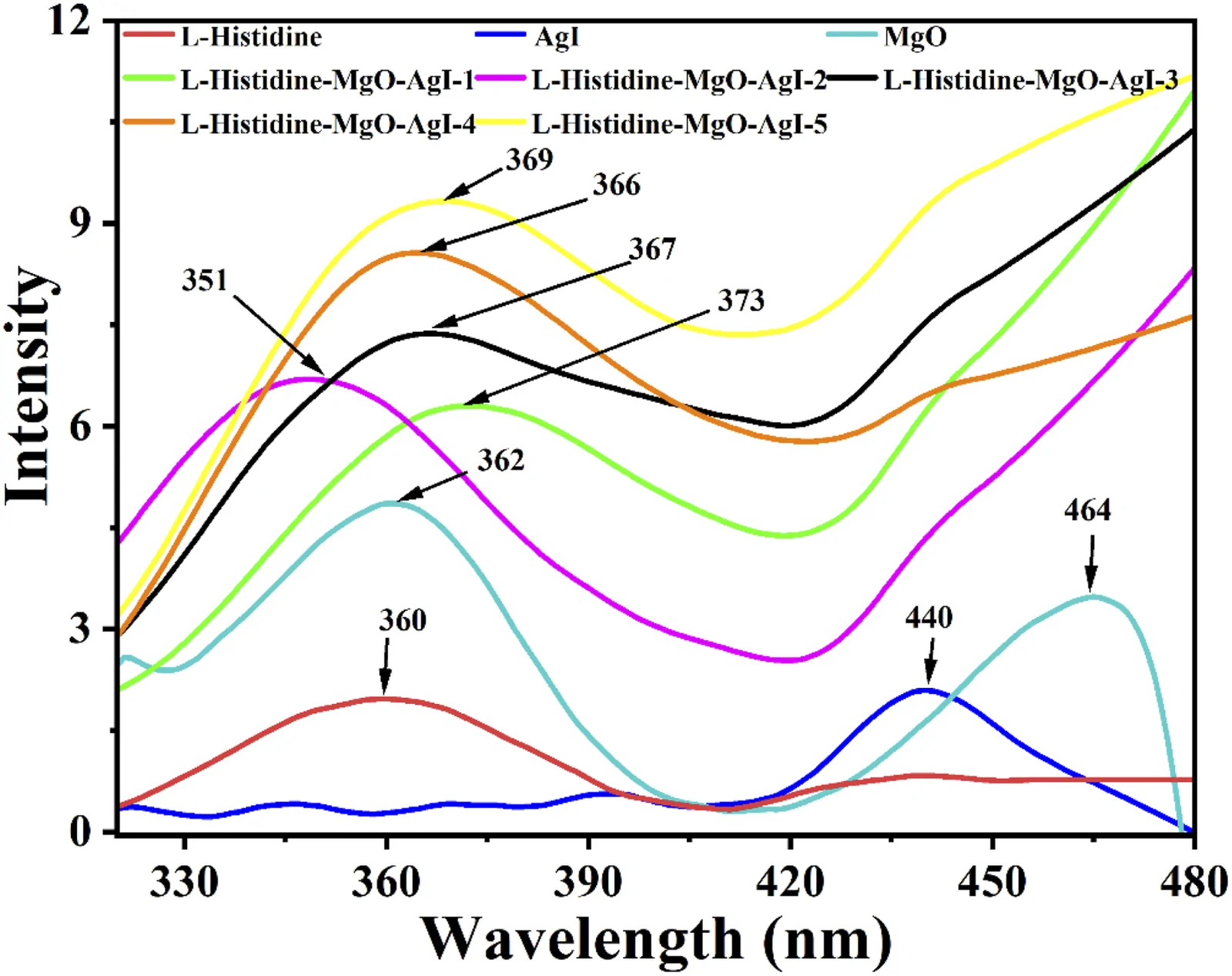
Photoluminescence spectra of l-histidine, MgO, AgI, and l-histidine–MgO–AgI composites.

Pure l-histidine exhibits an emission band at 355 nm under UV excitation, which is associated with an intramolecular electronic transition. The emission intensity may vary with molecular conformation and local environment (Fig. 6).^37^ In MgO, the emission peak at 362 nm is due to the direct recombination of holes and electrons in the energy band, while the visible emission band at 464 nm is caused by oxygen vacancies (Fig. 6).^38^ MgO shows significantly stronger emission than l-histidine because of its intrinsic luminescent properties. In AgI, the emission at 440 nm is due to radiative excitonic recombination (Fig. 6).^39^ Upon formation of the l-histidine–MgO–AgI composites, both the emission position and intensity were modified compared with the individual components (Fig. 6). The composite samples exhibited emission bands in the range of 351–373 nm with intermediate PL intensities relative to pure l-histidine and MgO. This intermediate emission behavior is consistent with the coexistence of the inorganic semiconductor and the organic ligand, together with interfacial interactions that modify the local electronic environment of the emitting species.

The calculated Stokes shifts for l-histidine, MgO, AgI, and the composite samples were 145, 198, 18, 161, 138, 153, 151, and 153 nm, respectively. These values indicate differences in the energy relaxation between photoexcitation and emission among the individual components and the composite materials. The intermediate Stokes shift values observed for the composites suggest that the emission characteristics are influenced by the combined contributions of l-histidine, MgO, and AgI, reflecting changes in the local electronic environment following composite formation.

### 3.4. Morphological topography

The morphological topography of l-histidine, and composite samples was attained from their FESEM micrographs (Fig. 7). The FESEM images of the composites obtained from the lowest and highest addition of MgO–AgI (l-histidine–MgO–AgI-1, l-histidine–MgO–AgI-5) are provided as a representative of all composites in Fig. 7b and c, respectively. From the micrographs, l-histidine exhibited aggregated, nonuniform sheet-like structures having a diameter of 100–600 nm (Fig. 7a). Similar morphological topography of l-histidine was noticed and discussed in ref. 40.

**Fig. 7:**
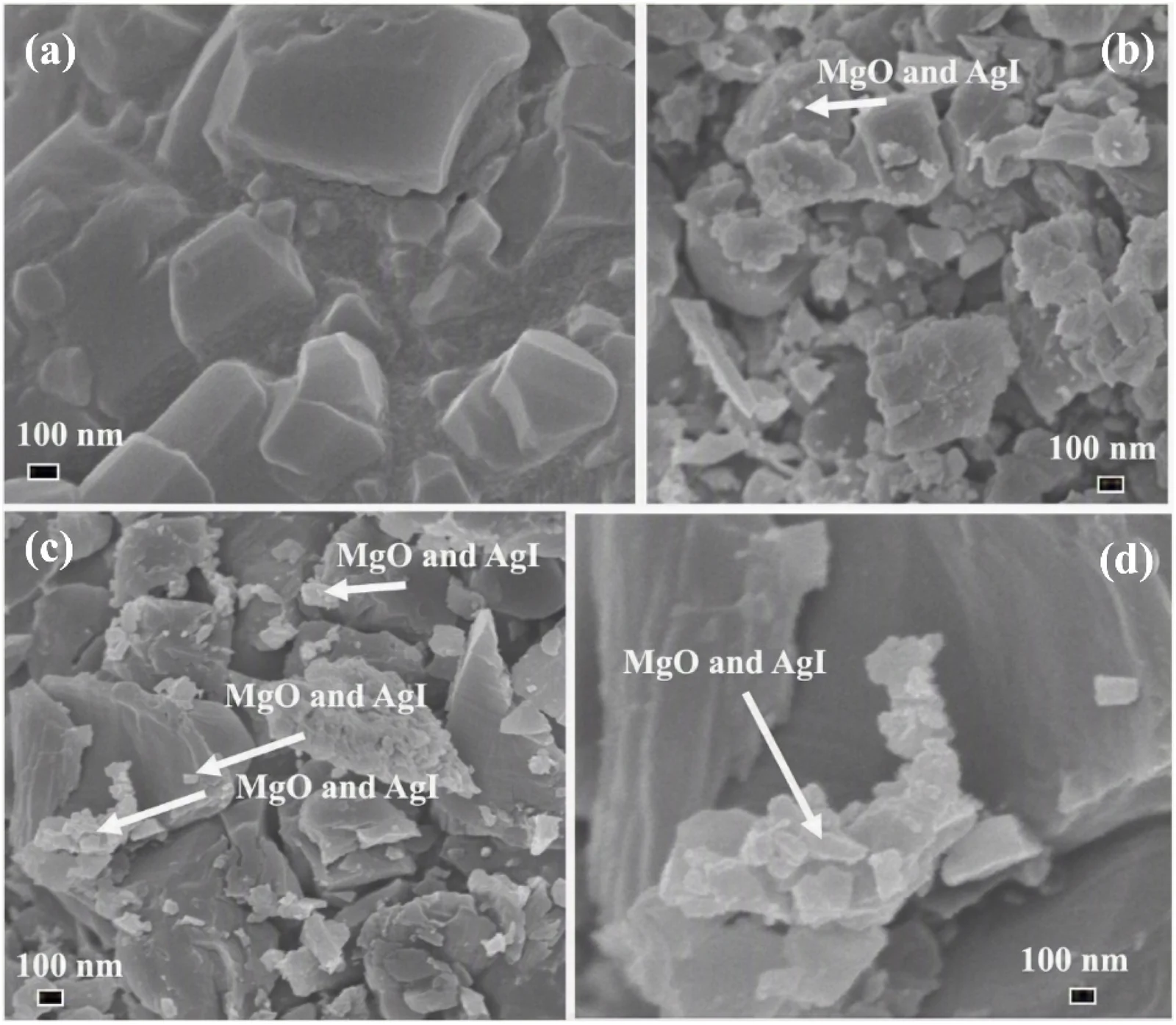
FESEM micrographs of (a) l-histidine, (b) l-histidine–MgO–AgI-1, and (c) l-histidine–MgO–AgI-5 composites. The white arrows in (b–d) show the existence of MgO–AgI structure in the composites, and (d) designate the higher magnification image of agglomerated MgO–AgI structure.

Concerning the l-histidine–MgO–AgI-1composites fabricated with the lowest addition of MgO–AgI (1 wt%), the structures are comprised of nonuniform with different-shaped including sheet-like structures like l- histidine morphology, and aggregated structure of MgO–AgI are visible (Fig. 7b). In this case, the diameter of the composites ranges from 50–600 nm. The structure of MgO–AgI is marked with white arrow, as can be seen in the Fig. 7b–d. However, the MgO–AgI structure is less visible for the composites obtained from the lowest addition of MgO–AgI. For the highest addition of MgO–AgI (10 wt%), the prepared composite structures remained same as l-histidine–MgO–AgI-1 composites, however, the diameter is slightly increased ranging from 50–800 nm (Fig. 7c). In this case, the MgO–AgI structure is more appreciated than the composites obtained with lowest addition of MgO–AgI. The higher magnification image of MgO–AgI is presented in Fig. 7d. Similar presence of materials in the amino acid based composite samples is reported in ref. 14 Consequently, by incorporating the suitable quantity of MgO–AgI, the morphological topography of the composites may be regulated within a diameter range of 50–800 nm. This morphological tendency significantly influences the photocatalytic activity of composites fabricated by incorporating MgO and AgI.^14,41^

To perceive more evidently the integration of MgO and AgI with l-histidine, EDX measurements were performed for the l-histidine–MgO–AgI composites. For example, Fig. 8 signifies the EDX spectrum of l-histidine–MgO–AgI-1 composites (1 wt% MgO–AgI addition). For l-histidine–MgO–AgI composites, not only the elements of C, N, O are identified, but also Mg, Ag, and I are observed. Such existence of Mg, Ag, and I in the composite's supplements sensibly with FTIR and UV-visible data and further confirm the formation of l-histidine–MgO–AgI composites. The atomic % of Mg, Ag and I in the composite sample are 0.61, 0.07 and 0.08, respectively as demonstrated in the inset of Fig. 8. Thus, the peaks recognized for Mg, Ag, and I in the spectrum confirm their integration in l-histidine–MgO–AgI. Like enclosure of inorganic and semiconducting materials in composite samples was previously stated in the literature.^14,41^

**Fig. 8:**
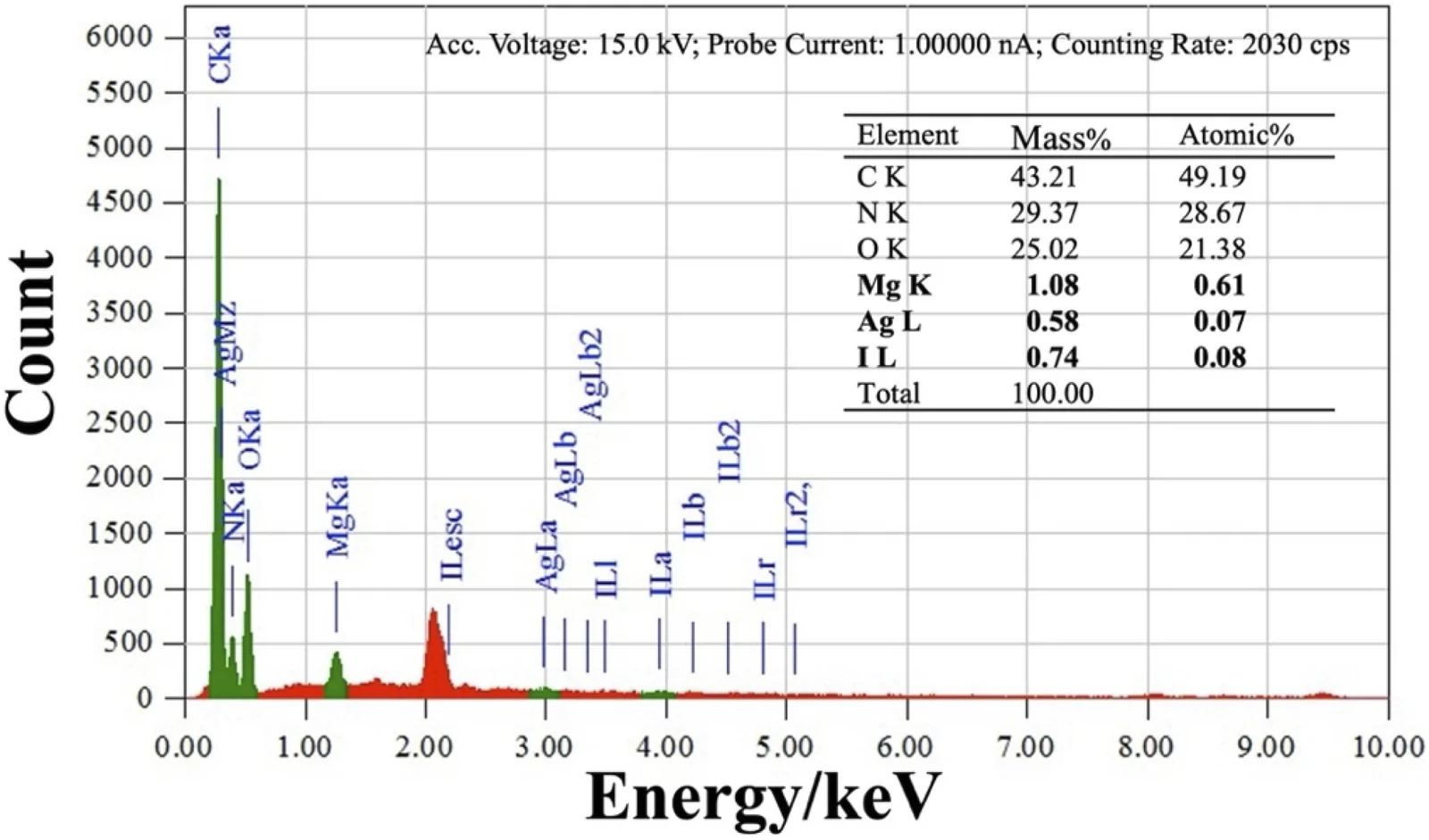
EDX spectrum of l-histidine–MgO–AgI-1 composites.

### 3.5. XRD analysis

The XRD patterns of pristine l-histidine, MgO, AgI and l-histidine–MgO–AgI-5 are shown in Fig. 9. Pristine l-histidine shows reflections at 15.3°, 18.96°, 21.11° and 24.27°, indexed to the (100), (110), (111) and (11–2) planes.^42,43^ MgO shows reflections at 38.1°, 43° and 62.3°, assigned to the (111), (200) and (220) planes of cubic MgO (JCPDS 01-075-0447).^44^ Similarly, the AgI pattern exhibits characteristic reflections at approximately 23.92°, 39.42°, and 46.47°, assigned to the (002), (110), and (112) planes, respectively (JCPDS 09-0374).^45^ The XRD pattern of l-histidine–MgO–AgI-5 shows reflections from all three components. The l-histidine peaks appear at 15.39°, 19.02°, 21.23° and 24.21°, while the weaker MgO and AgI peaks appear at higher angles, with the AgI (002) peak overlapping the l-histidine (11–2) peak near 24°. Slight shifts in the peak positions are observed in the composite compared with the pristine materials, which may be attributed to interaction among l-histidine, MgO and AgI. The sharp and intense diffraction peaks indicate good crystallinity of the prepared material. The coexistence of the characteristic diffraction peaks of MgO, AgI, and l-histidine confirms the successful formation of the l-histidine–MgO–AgI composite without detectable impurity phases. The average crystallite size of the composite, calculated using the Scherrer equation, was found to be 33.12 nm, indicating the nanocrystalline nature of the synthesized composite.^14^

**Fig. 9:**
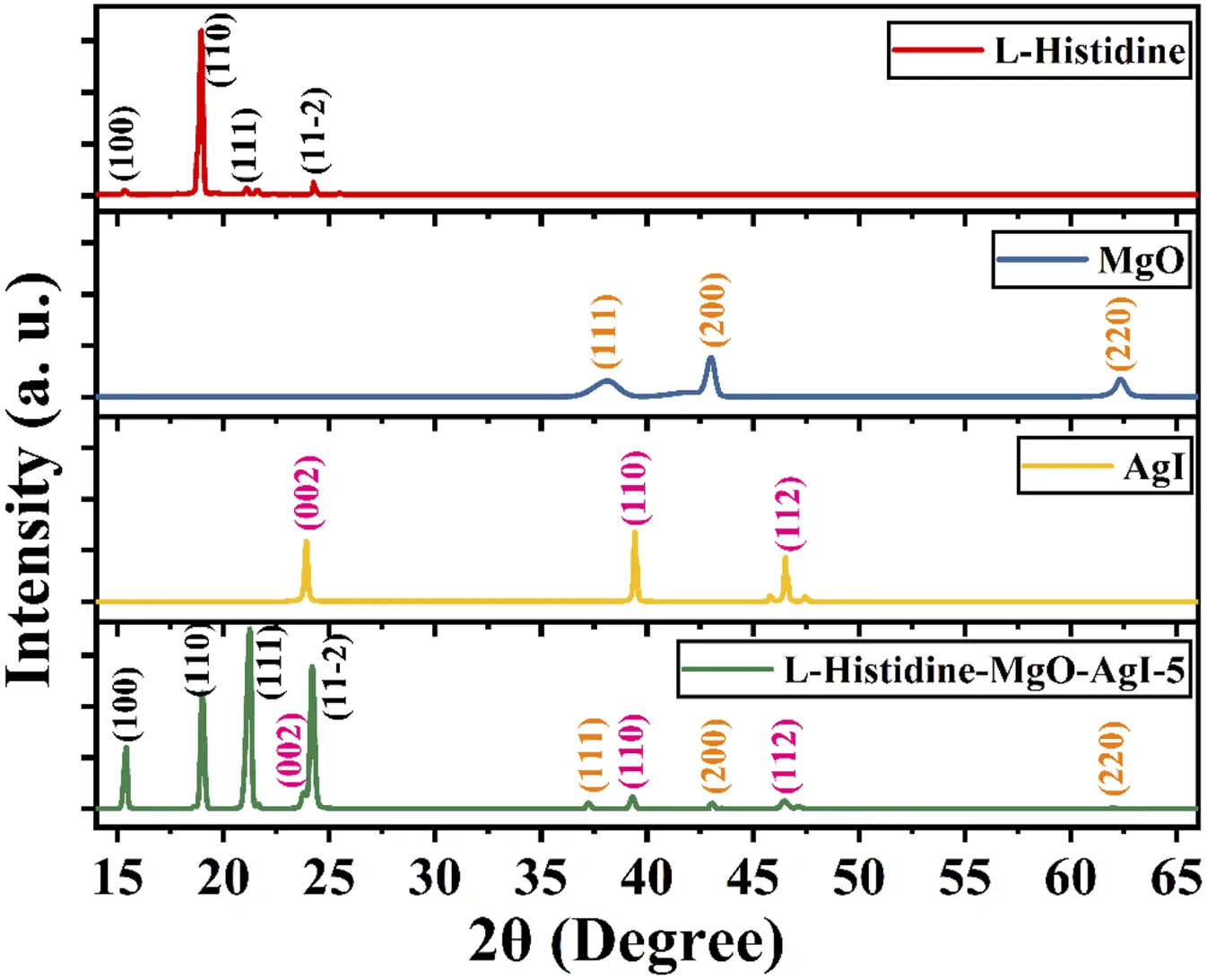
X-ray diffraction (XRD) pattern of the synthesized l-histidine–MgO–AgI-5 composite. The indexed *hkl* reflections corresponding to l-histidine, MgO, and AgI are shown in black, orange, and pink, respectively.

### 3.6. Thermogravimetric analysis (TGA)

Examining the thermal stability of materials is crucial for their proper applications. TGA tracks the change in sample mass as it is exposed to varying temperatures, capturing how a material responds to processes like evaporation, sublimation, decomposition, and oxidation.^25^ The thermal degradation properties of l-histidine and l-histidine–MgO–AgI-5 composite were examined using TGA, as presented in Fig. 10.

**Fig. 10:**
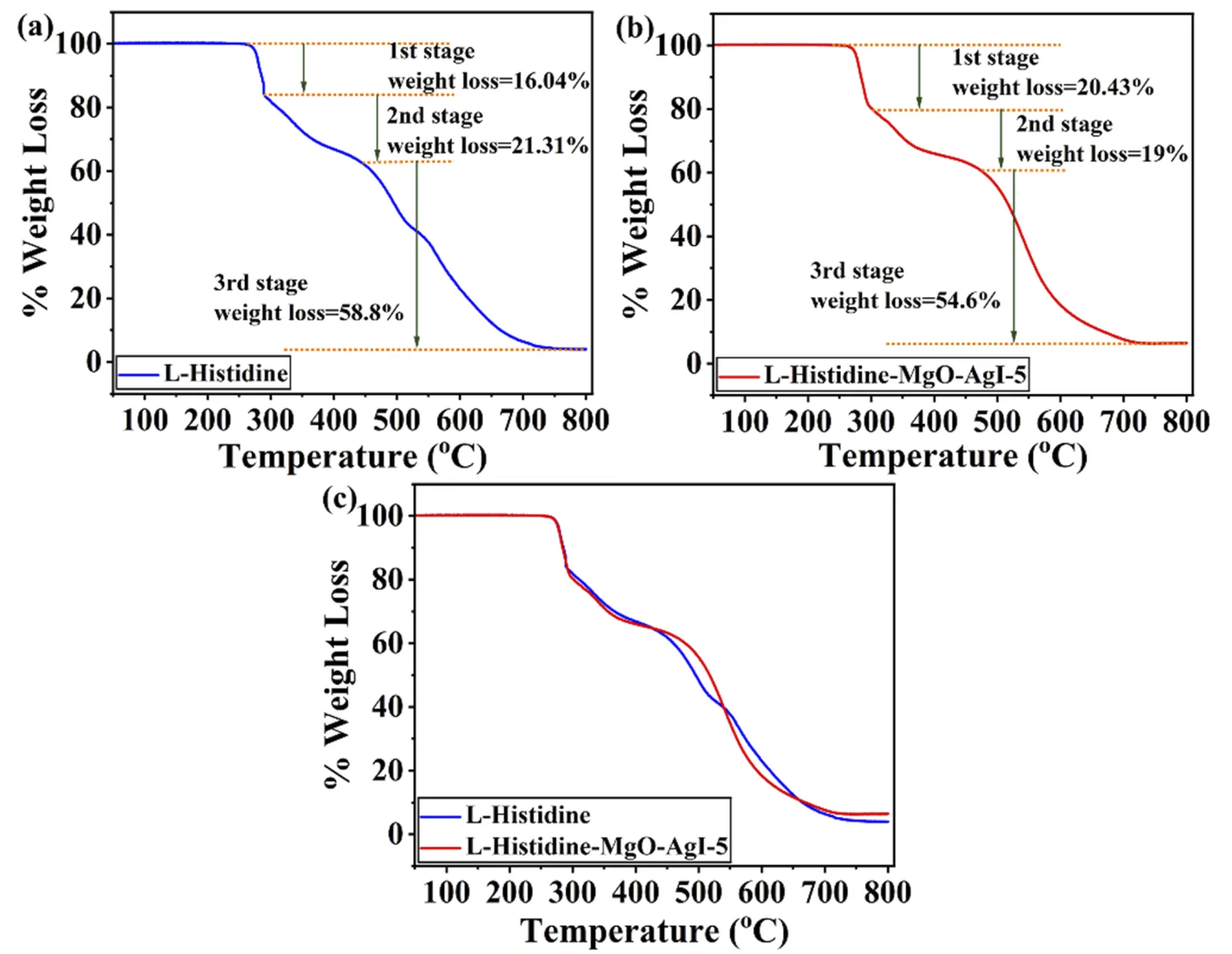
TGA curves of (a) l-histidine, (b) l-histidine–MgO–AgI-5 composite, and (c) l-histidine and l-histidine–MgO–AgI-5 composite.

Both l-histidine and the l-histidine–MgO–AgI composite exhibited a three-stage thermal degradation pattern, with distinct mass-loss events evident in their TGA curves (Fig. 10 and Table 3). The initial decomposition occurred at 267–306 °C for l-histidine and 264.5–305.8 °C for the composite (Table 3), corresponding to weight losses of 20.47% and 20.64%, respectively.^25^ This first thermal event is ascribed to the removal of weakly bound water molecules and the disruption of intermolecular hydrogen bonding within the histidine structure. The incorporation of MgO and AgI does not noticeably impact this phase since both substances can hold moisture on their surfaces, which evaporates when heated. The 2nd degradation stage was observed at 306.4–449.7 °C for l-histidine and 305.8–470.8 °C for the composite, with weight losses of 17.67% and 18.45%, respectively. This decomposition stage is associated with cleavage of the amino acid framework, including deamination, decarboxylation, and fragmentation of the imidazole ring. These processes result in the breakdown of functional groups (–NH_2_, –COOH) and evolution of volatile gases such as CO_2_ and NH_3_.^46^ The slightly higher degradation temperature range for the composite suggests that interaction between l-histidine and the MgO/AgI and able to restricts the molecular motion and increases the energy barrier for structural decomposition.^47^ The third major decomposition stage occurred between 449.7-729 °C for pure l-histidine and 470.8–712.8 °C for the composite, with mass losses of 58.62% and 54.73%, respectively.^25^ This stage corresponds to carbonization and oxidative decomposition of remaining organic residues. Notably, the composite shows a lower mass loss in this region, accompanied by a higher residual mass (6.18%) compared to pure histidine (3.24%).^48^ This indicates improved thermal stability of the composites due to the incorporation of thermally robust MgO and AgI phases, which act as stabilizing agents and inhibit the complete combustion of carbonaceous intermediates.

**Table 3:** Thermogravimetric analysis (TGA) of l-histidine and l-histidine–MgO–AgI-5 composite

Samples	Temperature (*T*) and % of weight loss (WL) at three decomposition stages						
	Stage 1		Stage 2		Stage 3		Residual weight (%)
	*T* (°C)	WL (%)	*T* (°C)	WL (%)	*T* (°C)	WL (%)	
l-Histidine	265–289	16.04	289–445	21.31	445–743	58.8	3.85
l-Histidine–MgO–AgI-5	255–303	20.43	303–475	19	475–721	54.6	5.97

### 3.7. Photocatalytic activity

The photocatalytic degradation of TC was investigated by observing changes in UV-vis absorption spectra with time in the presence of l-histidine, MgO, AgI, and all l-histidine–MgO–AgI composites, as represented in Fig. 11(a–h).

**Fig. 11:**
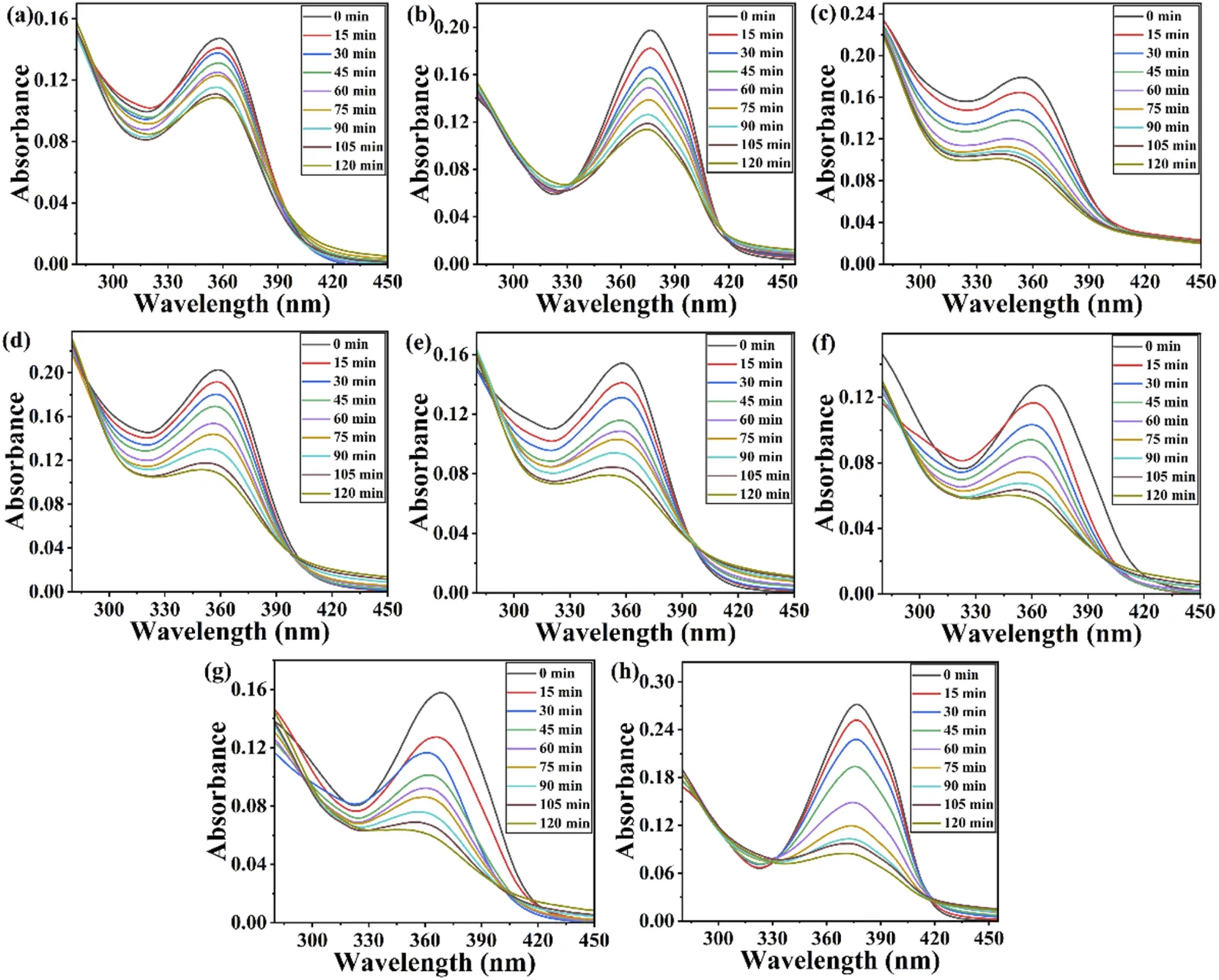
Variations in the UV-vis absorption spectra of the TC solution in the presence of (a) l-histidine, (b) MgO, (c) AgI, (d) l-histidine–MgO–AgI-1, (e) l-histidine–MgO–AgI-2, (f) l-histidine–MgO–AgI-3, (g) l-histidine–MgO–AgI-4, and (h) l-histidine–MgO–AgI-5; TC: 5 mg L^−1^ (100 mL), photocatalyst: 10 mg, pH: 7.

All experiments were conducted on the rooftop of Academic Building B at Shahjalal University of Science and Technology (SUST), Sylhet, Bangladesh (latitude: 24.9212986° N, longitude: 91.8328661° E). All experiments were performed on clear, sunny days (September, 2025; humidity: approximately 60%; cloud cover: 25–30%; temperature: around 35 °C) specifically during midday hours (11:00 AM to 1:00 PM) to ensure stable and maximal solar irradiation for consistent illumination across all samples. A gradual decrease in the absorption intensity of TC was perceived over time for all investigated photocatalysts, confirming the progressive degradation of TC molecules. The UV-vis spectra presented in Fig. 11b and c reveal that the absorption maxima of TC solutions containing 10 mg of bare MgO and AgI declined steadily with increasing irradiation time, corresponding to 42.42% and 43.33% removal efficiencies, respectively, after 120 min of sunlight exposure.^49^ On the other hand, l-histidine showed a relatively lower photocatalytic performance, achieving only 26.35% TC removal within the same period, indicating its limited ability to act as a photocatalyst under solar light (Fig. 11a). These findings underscore the superior sunlight-driven degradation efficiency of MgO and AgI compared to that of l-histidine. The photocatalytic activity of the l-histidine–MgO–AgI composites was further evaluated under identical solar irradiation conditions. As depicted in Fig. 11(d–h), a gradual decline in the absorption maxima of TC was observed over time, indicating the continuous degradation of TC.^50^ The consistent decrease in absorption intensity verifies the effective decomposition of TC during 120 min of solar irradiation.

As demonstrated in Fig. 12a, the l-histidine–MgO–AgI-1, l-histidine–MgO–AgI-2, l-histidine–MgO–AgI-3, l-histidine–MgO–AgI-4, and l-histidine–MgO–AgI-5 composites exhibited TC removal efficiencies of 44.83%, 48.05%, 52.71%, 59.49%, and 68.86%, respectively, after 120 min of solar exposure. The control experiment conducted under sunlight in the absence of a photocatalyst showed only 2.22% TC removal after 120 min, indicating that direct photolysis contributed negligibly to TC removal under the investigated conditions. These results demonstrate a marked improvement over the performance of l-histidine alone, confirming the enhanced photocatalytic activity of the composite materials. These results align well with previous reports on the removal of TC and other organic pollutants using MgO and AgI incorporated into composite structures.^51,52^ Compared to bare l-histidine, the l-histidine–MgO–AgI composites demonstrate superior photocatalytic activity towards the degradation of antibiotics such as TC. This improvement can be attributed to the pronounced synergistic interactions among l-histidine, MgO, and AgI in the composite, which enhance charge separation and reactive species generation under light irradiation. To assess the contribution of each component, binary composites were also evaluated (Fig. S1). MgO–AgI reached ∼68% removal after 120 min (*k* ≈ [value] min^−1^), comparable to the ternary composite, whereas l-histidine–MgO and l-histidine–AgI reached only ∼37% and ∼27%, below the pure components. These results indicate that the MgO–AgI interface is mainly responsible for the activity. Similar effects, where MgO and AgI are integrated with various materials (including polymers), have been reported to significantly boost the photocatalytic degradation of organic contaminants in aqueous solutions by other researchers.^11,52^ To investigate the effect of light irradiation, a control experiment was conducted in the absence of light, in which the l-histidine–MgO–AgI-5 composite exhibited only 2.67% TC removal after 120 min (Fig. S1).

**Fig. 12:**
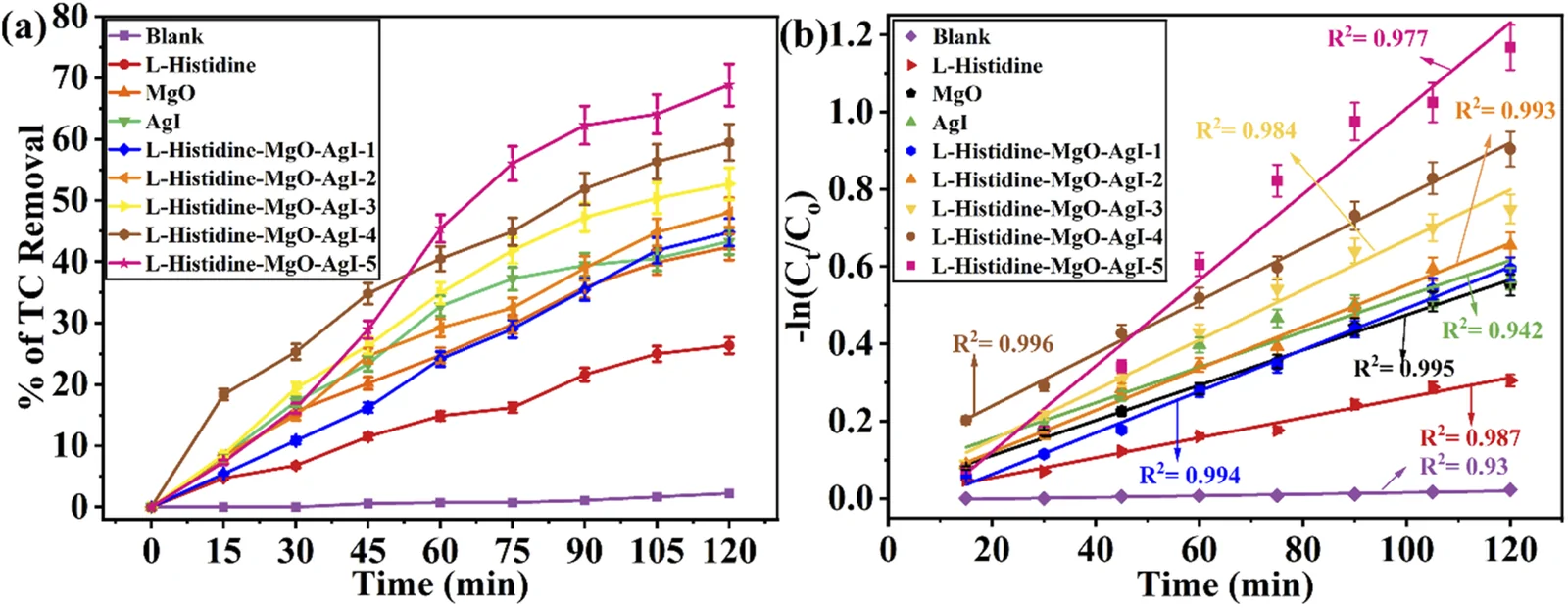
(a) % of TC removal with time, (b) pseudo-first-order kinetics model for TC removal of l-histidine, MgO, AgI, and all composite samples; TC: 5 mg L^−1^ (100 mL), photocatalyst: 10 mg, pH: 7.

To elucidate the kinetic behavior governing the photodegradation process of these composites, the variation of −ln(*C*_*t*_/*C*_o_) with irradiation time was examined, as depicted in Fig. 12b. The corresponding rate constants were determined by fitting the data to a pseudo-first-order kinetic model, as illustrated in the same figure. The pseudo-first-order kinetic fitting exhibited strong correlation coefficients, with *R*^2^ values between 0.942 and 0.996, validating the suitability and reliability of this kinetic model for describing TC degradation over the synthesized materials. Among them, the l-histidine–MgO–AgI-5 composite exhibited the highest rate constant of 11.11 × 10^−3^ min^−1^, followed by l-histidine–MgO–AgI-4 (6.81 × 10^−3^ min^−1^), l-histidine–MgO–AgI-3 (6.48 × 10^−3^ min^−1^), l-histidine–MgO–AgI-2 (5.4 × 10^−3^ min^−1^), and l-histidine–MgO–AgI-1 (5.37 × 10^−3^ min^−1^). The superior catalytic activity of the l-histidine–MgO–AgI-5 composite is evidenced by its higher rate constant in comparison to the other samples. Previous studies have also reported a similar trend in which the photocatalytic rate constant varies with the incorporation of different semiconducting components into polymer-based composites, corroborating these findings.^14^

To further investigate the photocatalytic activity of the l-histidine–MgO–AgI composite, additional tetracycline (TC) degradation experiments were conducted under UV-lamp (15 W, wavelength: 395 nm, model: LST-PAR-UV01, company: Okled) irradiation inside a chamber. The experimental parameters, including TC concentration, catalyst dosage, solution pH, and reaction volume, were kept consistent with those used in the sunlight experiments, while the irradiation source was changed. After 120 min of irradiation, the degradation efficiencies of l-histidine, MgO, AgI, l-histidine–MgO–AgI-1, l-histidine–MgO–AgI-2, l-histidine–MgO–AgI-3, l-histidine–MgO–AgI-4, and l-histidine–MgO–AgI-5 were 17.42%, 59.45%, 32.73%, 52.56%, 54.57%, 56.53%, 58.48%, and 67.04%, respectively. Among the investigated materials, l-histidine–MgO–AgI-5 exhibited the highest TC degradation efficiency of 67.04%. The photocatalytic degradation efficiencies of the starting materials and prepared composites under UV-lamp irradiation are represented in Fig. S2(i). The corresponding pseudo-first-order kinetic plots are presented in Fig. S2(j). The degradation kinetics followed the pseudo-first-order model, with the rate constants (*k*) and correlation coefficients (*R*^2^) for each condition summarized in Table S1 and S2.

### 3.8. HPLC analysis

To validate the photocatalytic degradation performance, the concentration of TC was determined using high-performance liquid chromatography (HPLC). The degradation process was investigated employing the l-histidine–MgO–AgI-5 composite as the photocatalyst, and the resulting changes in TC concentration were monitored by HPLC analysis. For quantitative analysis, an external calibration curve was established using TC standard solutions at concentrations of 4.0, 6.0, and 10.0 µg mL^−1^, yielding peak areas of 14 324, 19 149, and 22 538, respectively.^53^ Linear regression analysis demonstrated linearity (*R*^2^ = 0.9178) over the investigated concentration range, and the calibration equation was obtained as:

5 *y* = 1294.5*x* + 10041

where *y* represents the HPLC peak area and *x* represents the TC concentration (µg mL^−1^). This calibration equation (eqn 5) was subsequently employed to determine the residual concentration of TC after photocatalytic treatment. The initial TC concentration was 10.0 µg mL^−1^ at 0 min. Under the optimized conditions, the TC peak area (retention time = 2.235 min) fell from 22 538 at 0 min (10.0 µg mL^−1^) to 12 006 after 240 min of sunlight irradiation (Fig. S4). Using the calibration equation, the residual TC concentration was calculated to be 1.52 µg mL^−1^. Accordingly, the degradation efficiency was calculated to be 84.80%. This value is in good agreement with the removal efficiency of 83% obtained independently from UV-vis spectroscopy over the 240 min irradiation period (Fig. S4(a and b)). The close agreement between the two independent analytical techniques demonstrates the effective photocatalytic degradation of TC by the l-histidine–MgOyAgI-5 composite.^14,53^

### 3.9. Batch studies

#### 3.9.1. Concentration effect

The effect of the initial TC concentration on its removal by the l-histidine–MgO–AgI composite was investigated (Fig. 13a and b). The influence of the initial TC concentration on the photocatalytic enactment of the l-histidine–MgO–AgI-5 composite was examined at concentrations of 5 mg L^−1^, 10 mg L^−1^, 15 mg L^−1^, 20 mg L^−1^, and 25 mg L^−1^. The degradation rate declined with rising TC concentration, with removal efficiencies of 68.86%, 67.89%, 58.16%, 55.51%, and 53.73% observed after 120 minutes for the concentrations of 5 mg L^−1^, 10 mg L^−1^, 15 mg L^−1^, 20 mg L^−1^, and 25 mg L^−1^, respectively (Fig. 13a).

**Fig. 13:**
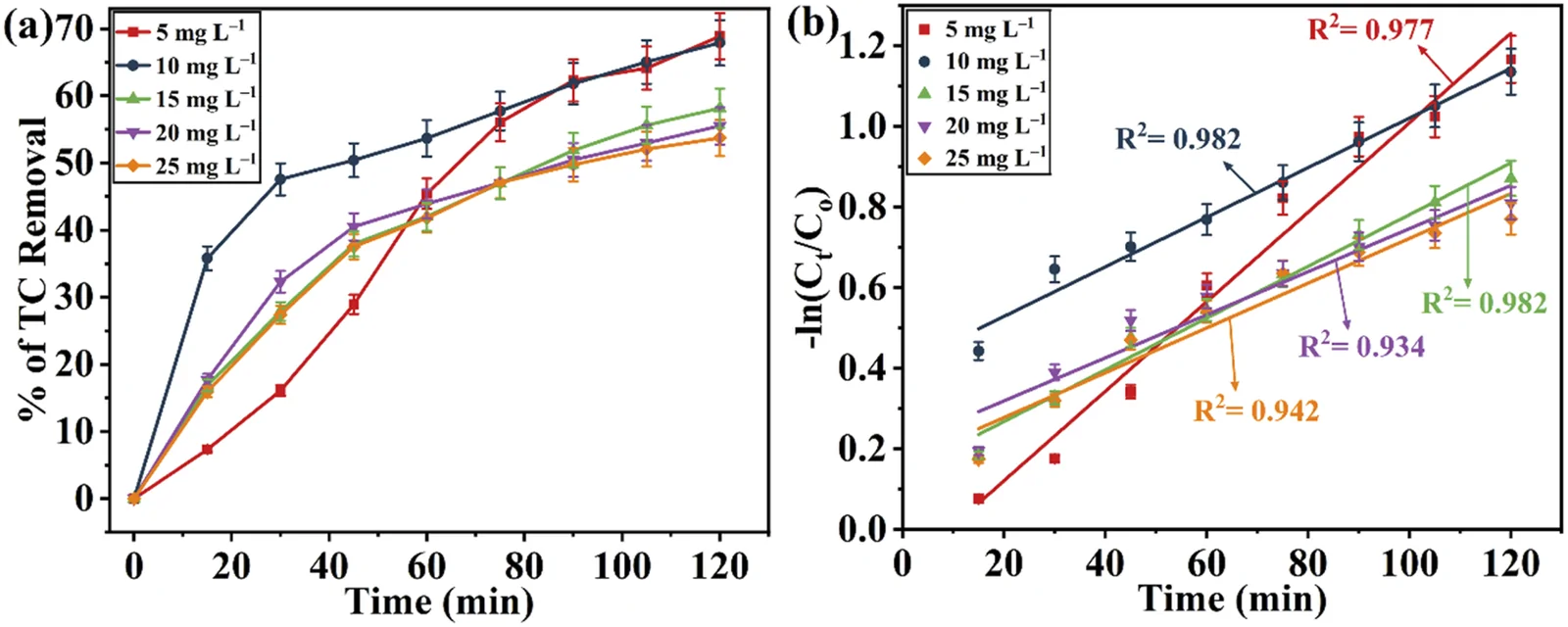
(a) Effect of concentration on percentage of TC removal, (b) pseudo-first-order kinetic model for TC removal at different concentrations (TC: 100 mL, photocatalyst: 10 mg of l-histidine–MgO–AgI-5 composite, pH: 7).

The findings reveal that increasing the TC concentration results in a noticeable decline in degradation performance. The removal efficiency decreased from 68.86% at 5 mg L^−1^ to 53.73% at 25 mg L^−1^ after 120 minutes of irradiation, demonstrating that the photocatalytic activity of the composite is strongly affected by the concentration of TC in the solution. The reduction in efficiency at higher concentrations can be mainly attributed to the reduced number of available active sites and decreased photon penetration.^54^ As more TC molecules are introduced into the system, they compete for adsorption on the limited number of active sites present on the catalyst surface, leading to site saturation.^55^ Simultaneously, the increased concentration of TC enhances light absorption within the solution, thereby limiting the transmission of photons to the photocatalyst surface.^56^ This light shielding effect suppresses the generation of photogenerated charge carriers and reactive oxygen species (˙OH and ˙O_2_^−^), which are crucial for effective degradation.^57^Fig. 13b shows the first-order kinetic model describing the effect of different TC concentrations on removal efficiency, with rate constants measured at 11.11 × 10^−3^ min^−1^,6.15 × 10^−3^ min^−1^, 6.42 × 10^−3^ min^−1^, 5.34 × 10^−3^ min^−1^, and 5.56 × 10^−3^ min^−1^for concentrations of 5 mg L^−1^, 10 mg L^−1^, 15 mg L^−1^, 20 mg L^−1^, and 25 mg L^−1^, respectively. Previous studies have established that higher rate constants correspond to more rapid antibiotic removal at specific concentrations. The model's fit for all composite samples is supported by the observed correlation coefficients (*R*^2^ values) provided in Fig. 13b.^22^ The correlation coefficients for 5 mg L^−1^, 10 mg L^−1^ and 15 mg L^−1^ were high, with *R*^2^ values ranging from 0.977 to 0.982, confirming that the TC degradation data fit well with the pseudo-first-order kinetic model.

#### 3.9.2. Dosage effect

The amount of catalyst used significantly affects the photocatalytic degradation efficiency.^56,58,59^ The impact of the initial TC concentration on its removal using the l-histidine–MgO–AgI-5 composite was examined, as illustrated in Fig. S5(a and b). TC was employed as a model pollutant to evaluate the photocatalytic activity at different catalyst dosages in a solution with a concentration of 25 mg L^−1^. Photocatalyst amounts of 5 mg, 10 mg, and 15 mg were tested, resulting in removal efficiencies of 38.13%, 53.73%, and 57.43%, respectively (Fig. S5(a)). The degradation efficiency of TC increases as the amount of catalyst rises because more catalyst provides a greater number of active sites for the adsorption and breakdown of TC molecules. With higher catalyst loading, there are more opportunities for the pollutants to interact with the catalyst, promoting the generation of reactive species needed for degradation. This improved availability of active sites enhances the overall photocatalytic process, resulting in increased removal of TC as the amount of catalyst is raised.^54,60^ Fig. S5(b) presents the plot of −ln(*C*_*t*_/*C*_o_) against irradiation time. The kinetic analysis reveals that the rate constant increases with catalyst dosage, with the highest value of 6.82 × 10^−3^ min^−1^, obtained at 15 mg. This is significantly greater than the rate constants observed at lower dosages of 5 mg (4.1 × 10^−3^ min^−1^), and 10 mg (5.56 × 10^−3^ min^−1^).^61^ For 5 mg and 15 mg, pseudo-first-order kinetic fitting yielded strong correlation coefficients (*R*^2^ = 0.997 and 0.967), validating the suitability and reliability of this kinetic model for describing TC degradation in the synthesized materials.

#### 3.9.3. pH effect

The photocatalytic study considering pH was conducted because pH influences both the surface charge and the electron transfer capability of the photocatalyst.^62^ The initial pH of the solution is crucial in the photocatalytic degradation of TC since the surface charges of both the TC molecule and the photocatalyst strongly depend on pH. This pH dependence affects the photocatalyst's interaction with TC and facilitates electron transfer, ultimately impacting degradation efficiency.^63^ The impact of pH of the initial TC concentration on its removal using the l-histidine–MgO–AgI-5 composite was examined, as illustrated in Fig. S6(a and b). The photocatalytic degradation of TC by the l-histidine–MgO–AgI composite was investigated to understand the effect of pH, examining both acidic and alkaline conditions. For this, the pH was adjusted to an acidic value of 4 using 0.1 mol L^−1^ HCl, and to an alkaline value of 10 using 0.1 mol L^−1^ NaOH, respectively.^60^ This setup allowed for studying how the initial pH influences the degradation process. According to Fig. S6(a), at an acidic pH of 4, TC removal efficiency is low, around 15.74% which further increases to 40.73% at neutral condition (pH 7). This is because, at very low pH, the excess H^+^ ions can neutralize crucial hydroxyl (OH^−^) and hydroxyl radicals (˙OH) needed for breaking down the antibiotic. In contrast, under alkaline conditions at pH 10, the degradation efficiency significantly improves to 63.37%, nearly four times higher than at acidic pH.^24^ Research indicates that TC degrades faster in alkaline environments due to nucleophilic attacks by hydroxide ions.^56^ The rate constant (*k*) at pH 4 is 1.25 × 10^−3^ min^−1^, which indicates delayed degradation, while at alkaline condition (pH 10), it rises to 8.14 × 10^−3^ min^−1^, showing accelerated degradation (Fig. S6(b)). This suggests alkaline conditions enhance photocatalytic degradation.^64^ At alkaline pH, TC mainly exists as an anion after deprotonation of functional groups like tricarbonyl or diketone phenol.^65^ The abundance of hydroxide ions helps generate reactive oxygen species (ROS), including hydroxyl and superoxide radicals, under light. These ROS more effectively degrade the anionic TC form compared to its neutral or cationic forms. The degradation route in alkaline medium involves reactions such as *N*-demethylation, deamination, hydroxylation, and dehydration, ultimately breaking TC down into smaller intermediates and mineralized products. The results showed that alkaline conditions favored TC degradation, with the highest removal efficiency observed at pH 10.^66^ Hence, it can be concluded that the synthesized catalyst is sensitive to variations in pH, showing its optimal activity in neutral to alkaline conditions. The kinetic plots at different pH levels showed excellent linearity, with *R*^2^ values of 0.986, 0.999, and 0.996 for pH 4, 7, and 10, respectively, confirming that TC degradation follows pseudo-first-order kinetics under all conditions.

#### 3.9.4. Interfering ion effect

The impact of interfering ions on the initial TC concentration on its removal using the l-histidine–MgO–AgI-5 composite was examined, as illustrated in Fig. 14a and b.

**Fig. 14:**
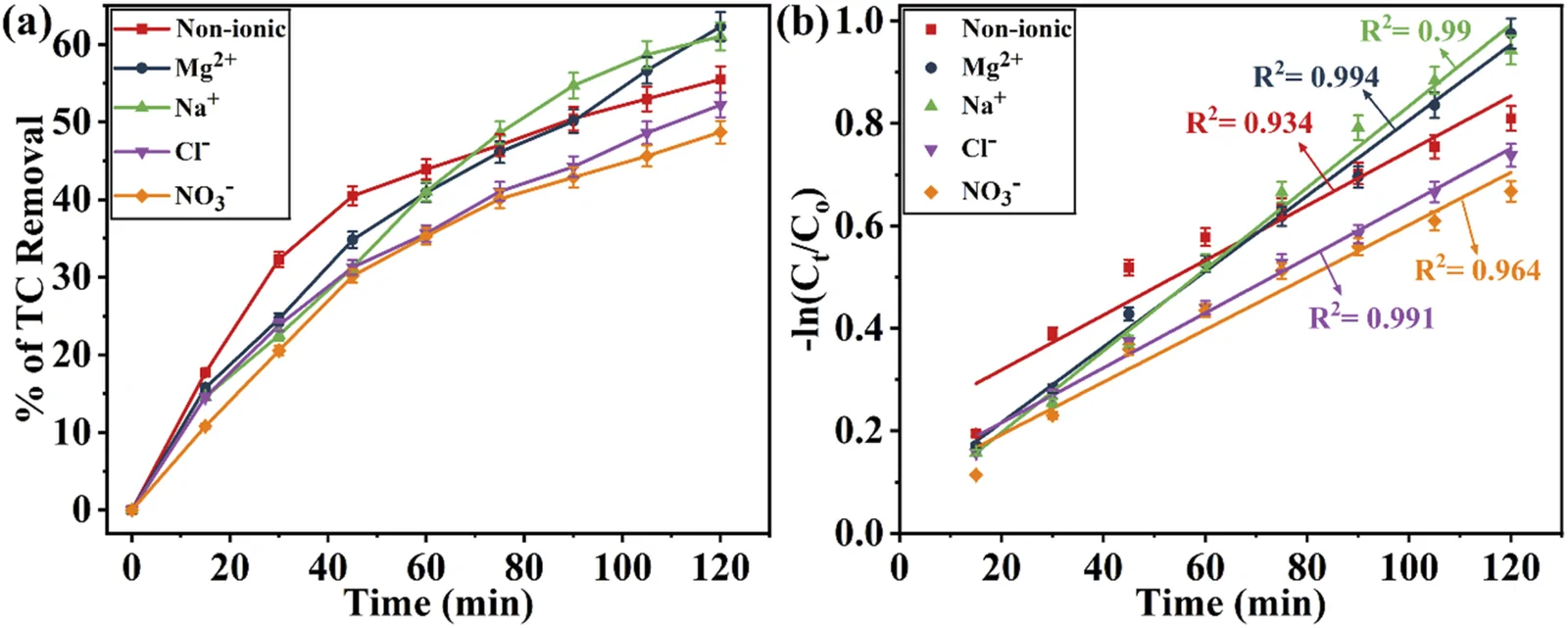
(a) Influence of interfering ion on percentage of TC removal, (b) pseudo-first-order kinetic model for TC removal at presence of different ions (TC: 20 mg L^−1^ (100 mL), photocatalyst: 10 mg of l-histidine–MgO–AgI-5, pH: 7).

To study the impact of interfering ions, TC solutions (20 mg L^−1^) containing MgSO_4_, Na_2_SO_4_, NaCl, and NaNO_3_ (each at 20 mmol L^−1^) were subjected to photocatalytic degradation using the l-histidine–MgO–AgI-5 composite under sunlight irradiation. The influence of Mg^2+^ and Na^+^ ions on photocatalytic activity was examined using Cl^−^ as the common anion. The experiments demonstrated that the presence of both Mg^2+^ and Na^+^ ions enhanced the degradation rate of TC, indicating that these cations positively affect the photocatalytic process.^57^ Under non-ionic conditions, the catalyst achieved 55.51% TC removal with a rate constant of 5.34 × 10^−3^ min^−1^, serving as the baseline. In presence of divalent cations (Mg^2+^), the degradation efficiency increased markedly to 62.31%, accompanied by a higher rate constant (7.37 × 10^−3^ min^−1^). The sharper decline in absorbance seen in Mg^2+^-containing spectra further supports this enhancement. Divalent ions tend to create stronger electrostatic interactions with the catalyst surface and pollutant molecules, improving adsorption and promoting more efficient charge separation, which collectively accelerate photocatalysis.^67^ The monovalent cation Na^+^ also enhanced TC degradation, achieving 61.06% removal with the highest rate constant (7.96 × 10^−3^ min^−1^). Although its effect is slightly lower than that of Mg^2+^, Na^+^ still improves surface charge distribution and upturns the accessibility of active sites, resulting in faster TC breakdown. Thus, both divalent and monovalent cations promote photocatalytic activity, with Mg^2+^ showing a stronger influence due to its higher charge density. Because a monovalent cation occupies a single adsorption site on the surface of l-histidine–MgO–AgI-5, whereas a Mg^2+^ ion occupies two vacant sites, thereby restricting the adsorption of TC molecules.^68^ The enhanced performance in the presence of Mg^2+^ is attributed to its stronger binding capacity with both the catalyst surface and the functional groups of TC, which improves adsorption and accelerates photocatalytic degradation.^57^ The influence of Cl^−^ and NO_3_^−^ ions on photocatalytic activity was examined using Na^+^ as the common cation. Anionic species (Cl^−^ and NO_3_^−^) interfered with TC degradation. Cl^−^ reduced the removal efficiency to 52.19%, while NO_3_^−^ resulted in the lowest activity (48.69% removal). This phenomenon occurs because these ions assimilate light photons, thereby hindering the photolysis of water molecules and limiting the formation of hydroxyl radicals. The presence of nitrate ions greatly decreases TC removal since these ions act as radical scavengers and generate less reactive oxidative radicals like 
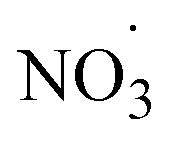
.^69^
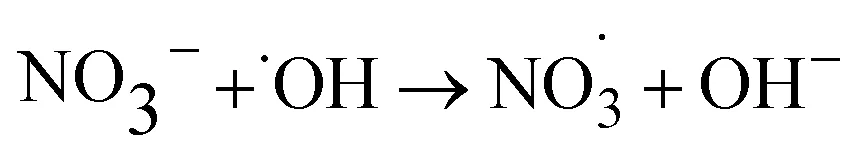


Chlorine ions readily react with hydroxyl radicals (˙OH) and positive holes (h^+^) on the surface of l-histidine–MgO–AgI-5, forming less reactive species.^70^ Additionally, the presence of chloride ions competes with pollutant molecules like TC for access to ˙OH radicals, limiting the pollutant's availability to these radicals. This competition partially explains the decreased photocatalytic efficiency observed when chloride ions are present.^71^Cl^−^ + h^+^ → ˙ClCl^−^ + ˙OH → ˙Cl + OH^−^Cl^−^ + ˙OH → ClOH˙^−^ClOH^−^ + H^+^ → ˙Cl + H_2_O˙Cl + Cl^−^ → Cl_2_˙^−^Cl_2_˙^−^ + Cl_2_˙^−^ → Cl_2_ + 2Cl^−^˙Cl + ˙Cl → Cl_2_Cl_2_ + H_2_O → HOCl + HClHOCl + H^+^ → OCl^−^

As these scavenging agents react with ˙OH radicals, causing a decline in their concentration and subsequently lowering the overall efficiency of the photocatalytic system.^72^ This results in slower absorbance decay in the corresponding spectra and lower rate constants (5.35 × 10^−3^ min^−1^ and 5.12 × 10^−3^ min^−1^, respectively). For the l-histidine–MgO–AgI-5 composite, the *R*^2^ values are consistently high (0.934–0.994), confirming an excellent linear fit and strong reliability of the calculated rate constants.

#### 3.9.5. Temperature effect

To understand the temperature dependence of the catalyst performance, a kinetic study was carried out using the l-histidine–MgO–AgI-5 composite, which revealed a distinct influence of thermal conditions on the photocatalytic degradation of TC. The impact of coexisting ions on the initial TC concentration on its removal using the l-histidine–MgO–AgI-5 photocatalyst was examined, as illustrated in Fig. S7(a and b). Since temperature is known to significantly affect photocatalytic reaction rates, the temperature-dependent degradation behavior of TC was investigated using the l-histidine–MgO–AgI-5 composite. The photocatalytic activity of the composite was tested at 40 °C, 50 °C, and 60 °C. As illustrated in Fig. S7(a), the percentage of TC removal increased steadily with the increase of temperature. Specifically, the degradation rate improved from 34.97% at 40 °C to 39.58% at 50 °C, and reaching 44.34% at 60 °C. Again, the rate constant for photocatalytic degradation typically increases with temperature due to enhanced reaction kinetics governed by the Arrhenius equation. Here, at 40 °C, the rate constant may be around 3.32 × 10^−3^ min^−1^, while at 50 °C, it rises to 3.78 × 10^−3^ min^−1^, reflecting faster reaction rates at higher temperatures (Fig. S7(b)). Further temperature increase can lead to an even higher rate constant, which is 4.61 × 10^−3^ min^−1^, indicating improved degradation efficiency.

The improvement in TC degradation at higher temperatures can be attributed to an increased frequency of collisions between TC molecules (or their intermediate forms) and the catalyst surface.^73^ This results in more efficient interactions at active sites where reactive hydroxyl radicals are produced. Additionally, elevated temperatures enhance the adsorption of TC molecules onto the catalyst, increasing the concentration of adsorbed species available for photocatalytic oxidation.^74^ Consequently, both the reaction rate and the overall degradation efficiency increase as the temperature increases.^75^ Moreover, the kinetic plots at different temperatures showed excellent linearity, with *R*^2^ values of 0.987, 0.959, and 0.996 for 40 °C, 50 °C, and 60 °C respectively, confirming that TC degradation follows pseudo-first-order kinetics under all conditions. Overall, the results demonstrate that increasing temperature accelerates the photocatalytic degradation of TC, which is consistent with Arrhenius behavior where higher thermal energy promotes faster reaction kinetics and more efficient formation of reactive radical species.^74^

#### 3.9.6. Effect of scavengers on TC degradation

To identify the reactive species responsible for TC degradation, scavenger experiments were carried out using isopropanol (IPA), disodium ethylenediaminetetraacetate (EDTA-2Na) and *p*-benzoquinone (BQ) as scavengers for ˙OH, h^+^ and ˙O_2_^−^, respectively (Fig. 15). In the absence of a scavenger, TC removal reached 67.88% after 120 min with a rate constant of 6.15 × 10^−3^ min^−1^. The addition of IPA, EDTA-2Na and BQ lowered the removal to 45.08%, 38.44% and 28.33%, and the rate constants to 4.22 × 10^−3^ min^−1^, 3.45 × 10^−3^ min^−1^ and 2.69 × 10^−3^ min^−1^, respectively. The strongest suppression was caused by BQ, showing that ˙O_2_^−^ is the dominant reactive species. EDTA-2Na also reduced the activity considerably, indicating that photogenerated holes make a significant contribution, while the smaller effect of IPA indicates that ˙OH plays a minor role. The relative contribution of these species follows the order ˙O_2_^−^ > h^+^ > ˙OH.^76^

**Fig. 15:**
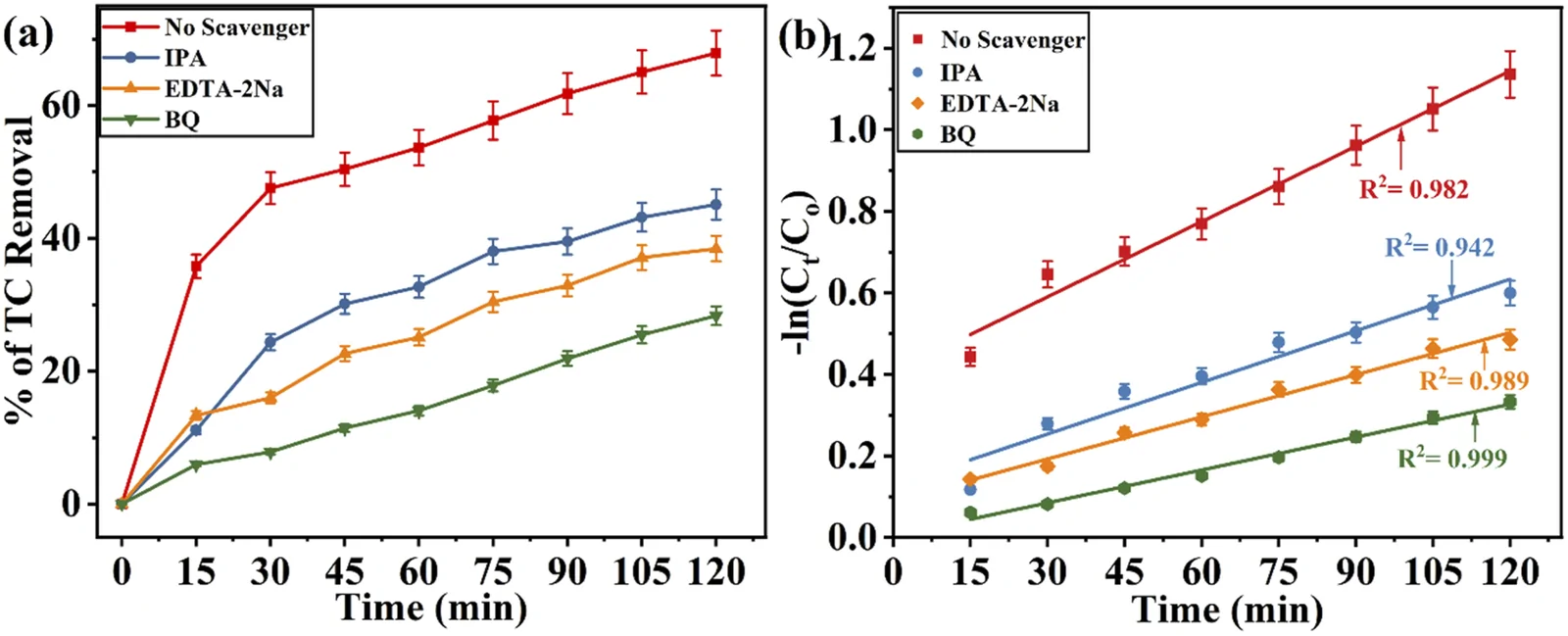
(a) Effect of radical scavengers on the percentage of TC removal under sunlight, (b) pseudo-first-order kinetic model for TC removal in the absence (control) and presence of IPA (˙OH), EDTA-2Na (h^+^) and BQ (˙O_2_^−^) (TC: 10 mg L^−1^ (100 mL), photocatalyst: 10 mg of l-histidine–MgO–AgI-5, scavenger: 1 mM, pH: 7).

### 3.10. Reusability study of l-histidine–MgO–AgI composite

The reusability of the synthesized l-histidine–MgO–AgI-5 photocatalyst was evaluated through consecutive photocatalytic degradation cycles of TC under identical experimental conditions. After each cycle, the catalyst was separated by centrifugation, thoroughly washed with deionized water, dried at 60 °C, and reused in the subsequent cycle without further treatment. The photocatalytic degradation efficiencies obtained during the recycling experiments are represented in Fig. 16. The photocatalyst retained excellent photocatalytic activity after three successive cycles, with only a slight decrease in degradation efficiency from 67.56% in the first cycle to 65.18% in the second cycle and 64.19% in the third cycle. This minor reduction may be attributed to the partial passivation of the catalyst surface caused by the adsorption of TC molecules, as well as the unavoidable loss of photocatalyst during the recovery and washing processes. Approximately 1.2 mg of catalyst was lost during each cycle. The FTIR spectra confirmed the structural stability of the l-histidine–MgO–AgI-5 composite after three successive photocatalytic cycles (Fig. 16b). Similar reusability behavior has also been reported for other composite photocatalysts in previous studies.^11,77^

**Fig. 16:**
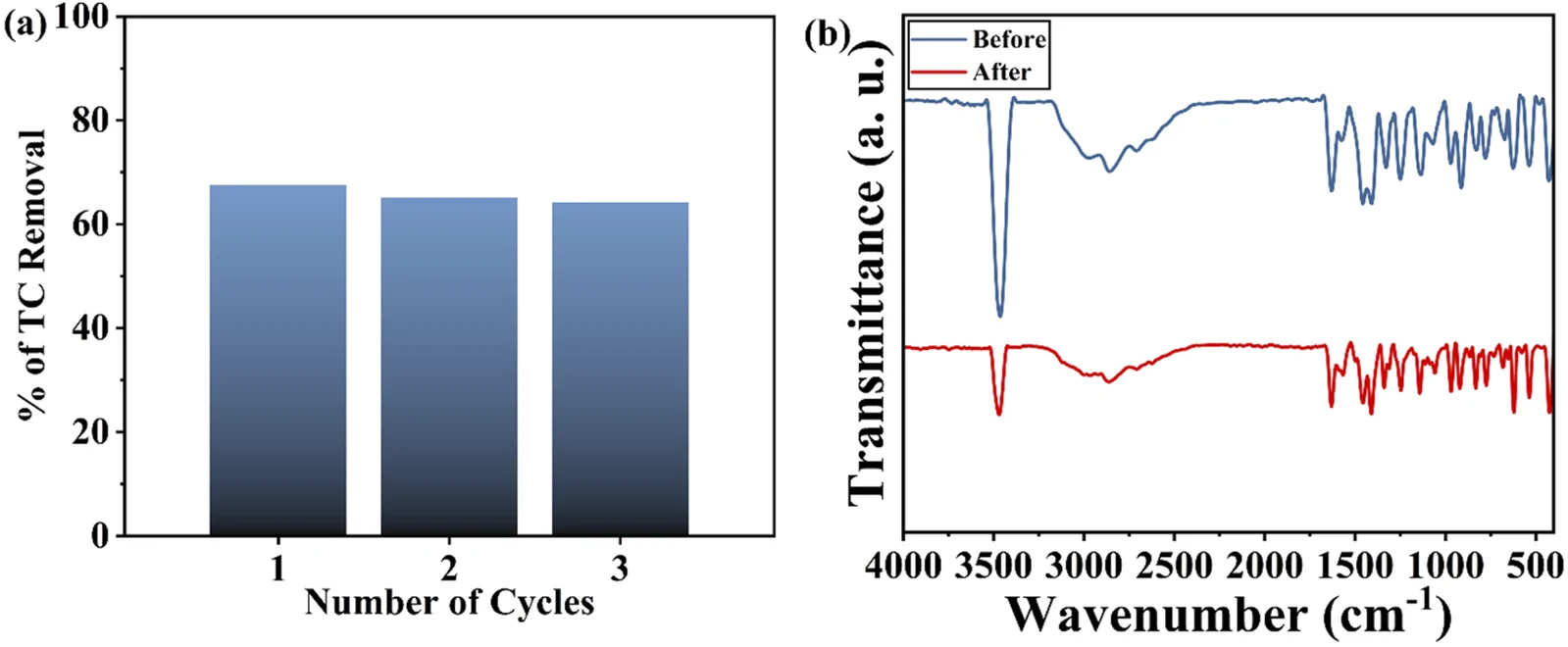
(a) Photocatalytic reusability of the l-histidine–MgO–AgI-5 composite for TC degradation during three successive cycles (initial TC concentration: 15 mg L^−1^ (100 mL), catalyst: 15 mg of l-histidine–MgO–AgI-5, pH: 7); (b) FTIR spectra of the l-histidine–MgO–AgI-5 composite before and after three photocatalytic degradation cycles.

### 3.11. Mineralization studies

TOC analysis was performed to evaluate the mineralization efficiency of the photocatalytic process. The l-histidine–MgO–AgI-5 photocatalyst achieved a mineralization efficiency of 38.23% after 120 min of visible-light irradiation. The lower mineralization efficiency compared with the photocatalytic degradation efficiency (68.86%) indicates that complete mineralization was not achieved within the reaction time. This difference can be attributed to the formation of intermediate organic species during the degradation process, which require further oxidation before being converted into CO_2_ and H_2_O. Therefore, a high degradation efficiency does not necessarily correspond to complete mineralization of the pollutant.^65^

### 3.12. Possible photocatalytic mechanism of TC degradation

Based on the roles of photogenerated electrons (e^−^), holes (h^+^), and reactive oxygen species (ROS), a possible mechanism for the photocatalytic degradation of tetracycline by the l-histidine–MgO–AgI composite under sunlight is proposed. The relative band positions of MgO and AgI may affect the movement of electrons and holes and the formation of reactive species, thereby influencing the photocatalytic activity of the composite. The conduction band (CB) and valence band (VB) potentials of the semiconductors were estimated using the Butler–Ginley equations (Eqn 6 and 7).^78^

6 *E*_VB_ = *χ* − *E*_e_ + 0.5 *E*_g_

7 *E*_CB_ = *E*_VB_ − *E*_g_

Here, *E*_VB_, *E*_CB_, and *E*_g_ represent the valence-band potential, conduction-band potential, and band-gap energy of the semiconductor, respectively, while *χ* denotes the absolute electronegativity of the semiconductor and *E*_e_ is the energy of free electrons on the hydrogen scale (≈4.5 eV).^78^

The band gaps of MgO and AgI were obtained from the Tauc plots (Fig. 5) as 4.17 and 2.79 eV, respectively, and *χ* values of 5.70 eV (MgO) and 5.48 eV (AgI) were taken from the literature.^78,79^ The calculated *E*_VB_ and *E*_CB_ are +3.29 and −0.89 V (*vs.* NHE) for MgO and +2.38 and −0.42 V for AgI. l-histidine is an organic component, so the Butler–Ginley equations do not apply to it. The band positions are shown in Fig. 17. The band positions were estimated for MgO and AgI only, as the Butler–Ginley equations apply to inorganic semiconductors.

**Fig. 17:**
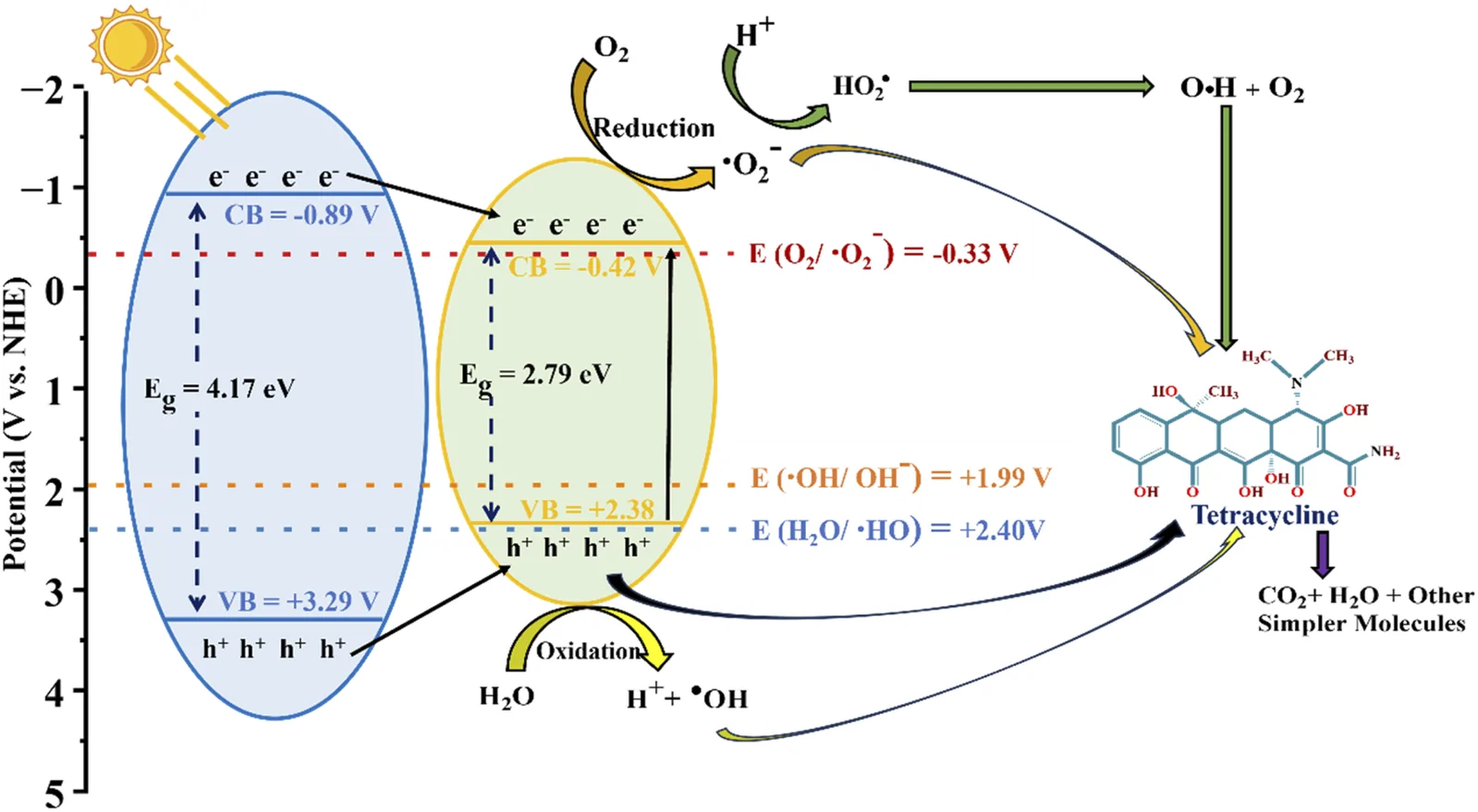
The proposed schematic illustration depicts the possible photocatalytic mechanism responsible for the degradation of TC under sunlight irradiation.

Under sunlight, AgI is excited across its 2.79 eV band gap, and electrons move from its VB to its CB, leaving holes in the VB. MgO showed clear photocatalytic activity under sunlight (Fig. 12). Because the CB (−0.89 V) and VB (+3.29 V) of MgO lie outside those of AgI (−0.42 and +2.38 V), electrons and holes generated in MgO can transfer to the CB and VB of AgI, respectively, since both transfers are energetically favorable.^80^ Together with the direct excitation of AgI, this leads to an accumulation of carriers on AgI, which corresponds to a straddling (type-I) alignment.^81,82^

The CB of AgI (−0.42 V) is more negative than E(O_2_/˙O_2_^−^) (−0.33 V), so the accumulated electrons can reduce adsorbed O_2_ to ˙O_2_^−^. The VB of AgI (+2.38 V) is more positive than E(˙OH/OH^−^) (+1.99 V) and close to E(H_2_O/˙OH) (+2.40 V), so holes can oxidize TC directly and can form ˙OH only to a limited extent. This agrees with the scavenger experiments (Fig. 15). l-histidine may further help by providing adsorption sites for TC.^83^ The reactive species finally degrade TC into intermediates and, possibly, CO_2_, H_2_O, and other small molecules.^84^Fig. 17 illustrates a likely mechanism for the photocatalytic degradation process of TC using l-histidine–MgO–AgI composites.

Ultimately, TC is decomposed into carbon dioxide (CO_2_), water (H_2_O) and other degradation products under light exposure.^85^ The MgO and AgI coating enhances TC adsorption on the active photocatalyst surface, making the l-histidine–MgO–AgI composite more effective than l-histidine alone. The sequence of reactions involved in the degradation pathway is illustrated by the following equations.^11,56^

i.Light-induced charge separationComposite + h*ν* → composite (e^−^_CB_ + h^+^_VB_)

ii.Generation of superoxide radicalse^−^_CB_ + O_2_ → ˙O_2_^−^

iii.Production of hydroxyl radicalsh^+^_VB_ + H_2_O → ˙OH + H^+^

iv.Formation and breakdown of hydrogen peroxide˙O_2_^−^ + 2H^+^ → H_2_O_2_H_2_O_2_ + e^−^ → ˙OH + OH^−^OH^−^ + h^+^ → ˙OH

v.Final mineralization of pollutants˙O_2_^−^ + ˙OH + TC → degradation products

### 3.13. Analytical identification of degradation intermediates

The formation of intermediate products during the photocatalytic degradation of TC was investigated using LC-MS analysis. The LC-MS spectra obtained after photocatalytic degradation (68.86%) showed several characteristic peaks corresponding to degradation intermediates (some of which could not be identified). The parent TC molecule exhibited a characteristic peak at *m*/*z* = 445 with a retention time of 3.32 min. The detected intermediates, together with their corresponding *m*/*z* values and proposed structures, are summarized in Table 4 and Fig. S8. The identified intermediates indicate that TC degradation initially involved transformations of peripheral functional groups, including deamination, deamidation, and dealkylation, while preserving the tetracyclic core structure.^86^ Further transformation of the detected intermediates likely involved ring opening and fragmentation of the TC skeleton, leading to the formation of lower-molecular-weight products.^87^

**Table 4:** Possible intermediate products identified during the photocatalytic degradation of TC based on LC-MS analysis

Intermediate products	*m*/*z*	Proposed structure
Tetracycline	445	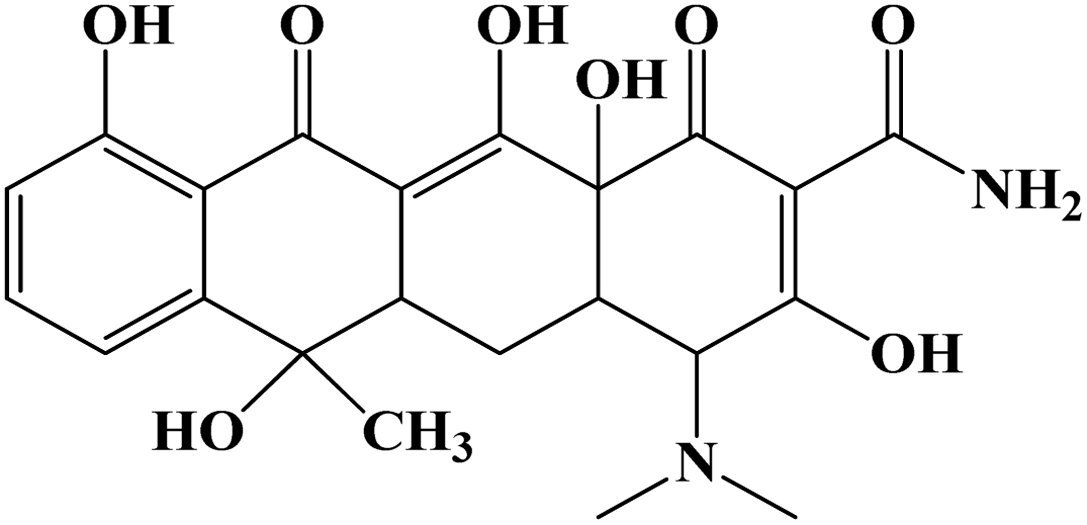
1	429	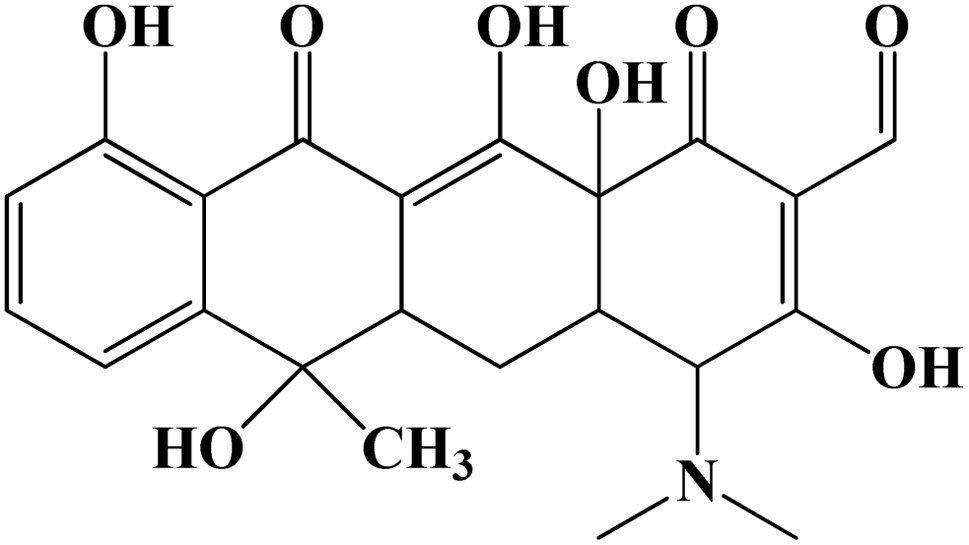
2	413	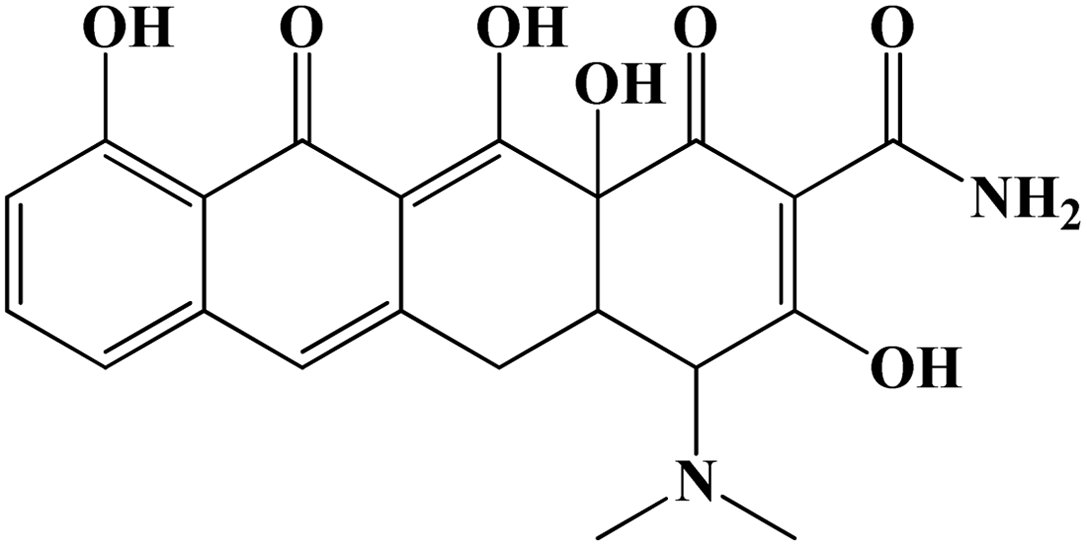
3	372	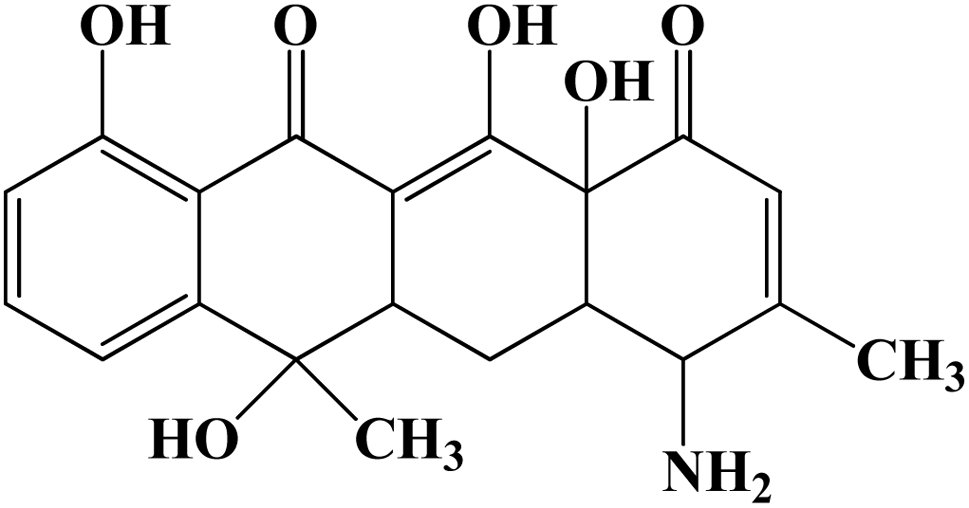
4	354	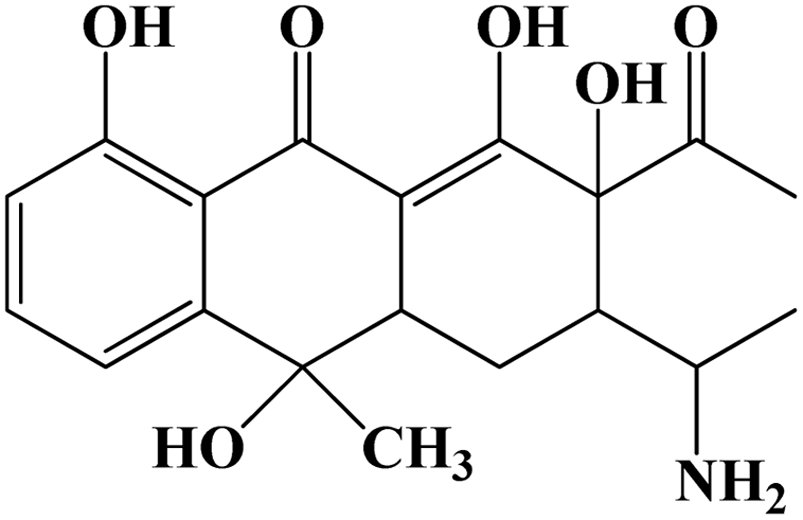
5	339	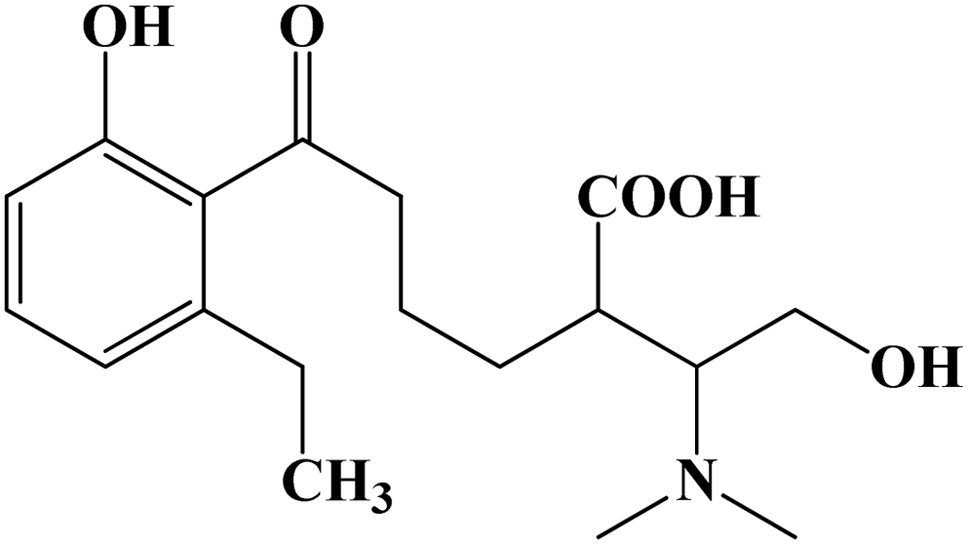
6	301	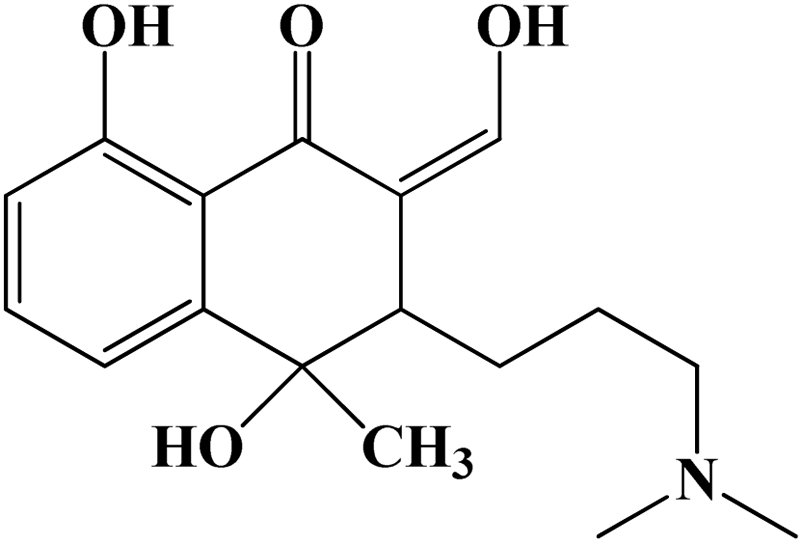
7	279	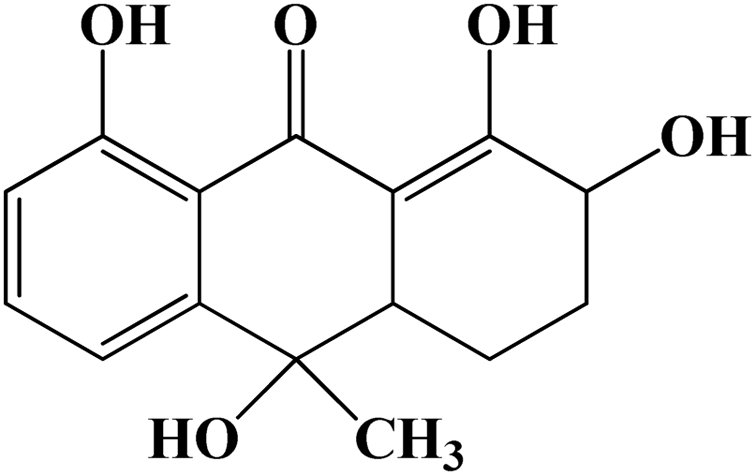
8	192	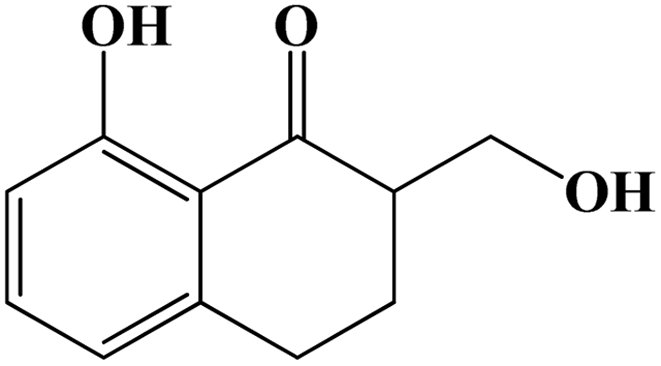
9	174	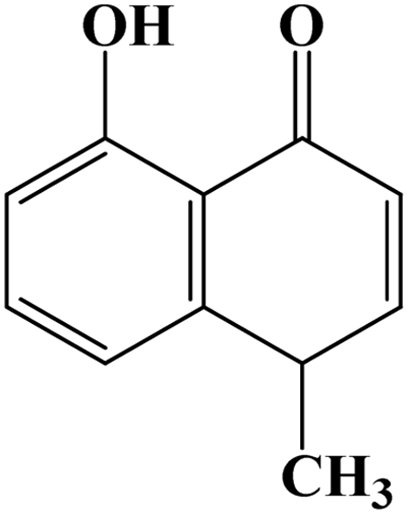
10	150	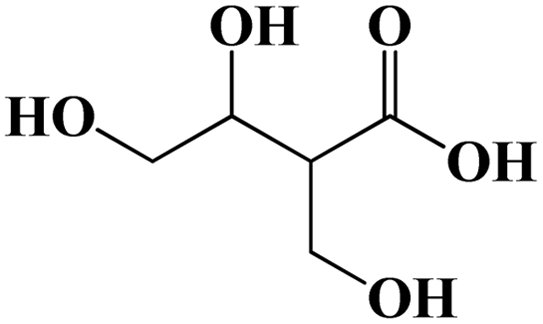

### 3.14. Comparative study

A comparison of the photocatalytic performances of different photocatalysts containing l-histidine, MgO, or AgI is presented in Table 5. As shown in Table 5, different catalyst dosages and initial TC concentrations were employed in the reported studies, leading to considerable variations in the photocatalytic degradation efficiency. Nevertheless, the synthesized l-histidine–MgO–AgI composite exhibited promising photocatalytic performance, demonstrating its potential for TC degradation. The improved performance can be attributed to the synergistic integration of the MgO–AgI heterojunction with l-histidine, which enhances pollutant adsorption, facilitates interfacial charge transfer, suppresses electron–hole recombination, and improves the stability of the photocatalyst. These features make the synthesized composite a promising candidate for TC degradation.

**Table 5:** Comparative photocatalytic performance of the synthesized l-histidine–MgO–AgI composite and previously reported l-histidine-, MgO-, and AgI-based photocatalysts for TC degradation

Photocatalyst	Target pollutant	Photocatalyst amount (mg L^−1^)	Initial pollutant concentration (mg L^−1^)	Time (min)	Efficiency (%)	Reference
l-Histidine–TiO_2_	Oxytetracycline	100	50	60	100	88
S–MgO	TC	12 000	50	10	99	89
AgI/BiVO_4_	TC	—	100	90	81	90
g-C_3_N_4_/BiOI/AgI	TC	400	—	165	91.8	91
AgI/ZnO	TC	1000	10	90	89.38	92
l-Histidine–MgO–AgI	TC	100	5	120	68.86	This study

## 4. Conclusion

l-Histidine–MgO–AgI composites were successfully synthesized by varying the MgO and AgI contents from 1 to 10 wt%. Spectroscopy data along with the morphological and thermal characterizations confirmed the effective incorporation of MgO and AgI into the l-histidine. FTIR, UV-vis, PL, FESEM, EDX, XRD, and TGA analyses collectively verified the formation of l-histidine–MgO–AgI composites with distinct emission features, heterogeneous microstructures, and improved thermal stability compared with pure l-histidine. FESEM images showed irregular, aggregated, and sheet-like structures with diameters of 50–800 nm, compared with 50–600 nm for pristine l-histidine. XRD analysis confirmed the crystalline nature of the composite, with an average crystallite size of 33.12 nm. TGA analysis demonstrated that incorporating MgO and AgI into l-histidine reduced the mass loss during the second thermal decomposition stage from 58.8% for pristine l-histidine to 54.6% for the composite, indicating improved thermal stability. The photocatalytic performance of the composites toward TC degradation was systematically investigated under sunlight by varying TC concentration, catalyst dosage, pH, temperature, coexisting ions, and scavengers. The composite containing 10 wt% MgO and AgI exhibited the highest removal efficiency of 68.86% at a TC concentration of 5 mg L^−1^, while 63.37% removal was obtained under alkaline conditions. Under UV-lamp irradiation, l-histidine–MgO–AgI-5 achieved 67.04% TC removal. Scavenger experiments identified ˙O_2_^−^ as the dominant reactive species, followed by h^+^ and ˙OH. Coexisting cations and anions were found to influence degradation efficiency, indicating that ionic species in solution affect the interactions among the catalyst surface, reactive species, and TC molecules. HPLC analysis indicated approximately 84.80% TC degradation after 240 min of sunlight irradiation. The composite retained a degradation efficiency of 64.19% after three consecutive photocatalytic cycles, while TOC analysis revealed 38.23% removal. Furthermore, LC-MS analysis was employed to identify the degradation intermediates. Overall, these findings indicate that the l-histidine–MgO–AgI composite is an effective photocatalyst for TC degradation under the investigated experimental conditions. Further studies involving real wastewater matrices, long-term performance evaluation, and process optimization are required to better assess its potential for practical wastewater treatment applications.

## Author contributions

Bristi Dutta investigation, methodology, validation, data curation, writing – original draft. Mohammad Mizanur Rahman Khan conceptualization, methodology, investigation, supervision, validation, writing – review & editing. Jae-Ho Jeong investigation, validation, data curation, writing – review & editing.

## Conflicts of interest

The authors declare that they have no known competing financial interests or personal relationships that could have appeared to influence the work reported in this paper.

## Supplementary Material

RA-OLF-D6RA08521K-s001

RA-OLF-D6RA08521K-s002

## Data Availability

The data used to support the findings of this study are available from the corresponding authors upon request. Supplementary information (SI) is available. See DOI: https://doi.org/10.1039/d6ra08521k.
