# Supplementary material for: Sunlight-driven removal of tetracycline over l-histidine–MgO–AgI composites: synthesis, morphological insight, and enhanced thermal stability
Source: RSC Adv. 2026 Nov 4. Online ahead of print. doi: 10.1039/d6ra08521k (PMC13732480; doi:10.1039/d6ra08521k)
Supplement: Supplementary file 1 [file RA-OLF-D6RA08521K-s002.pdf]

Supplementary Information (SI) for RSC Advances.
This journal is © The Royal Society of Chemistry

Supplementary materials

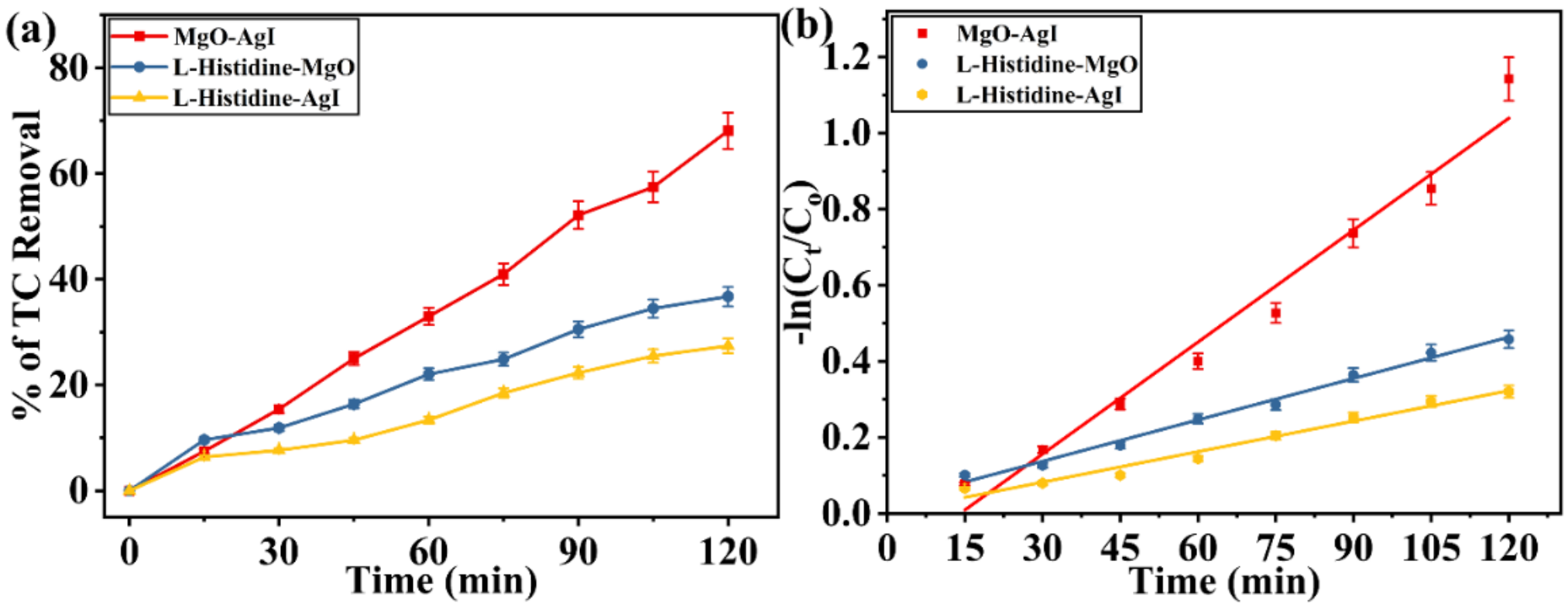

Fig. S1. (a) % of TC removal with time, (b) Pseudo-first-order kinetics model for TC removal of MgO-AgI, L-Histidine-MgO, L-Histidine-AgI; TC: 5 mg L^{-1} (100 mL), photocatalyst: 10 mg, pH: 7.

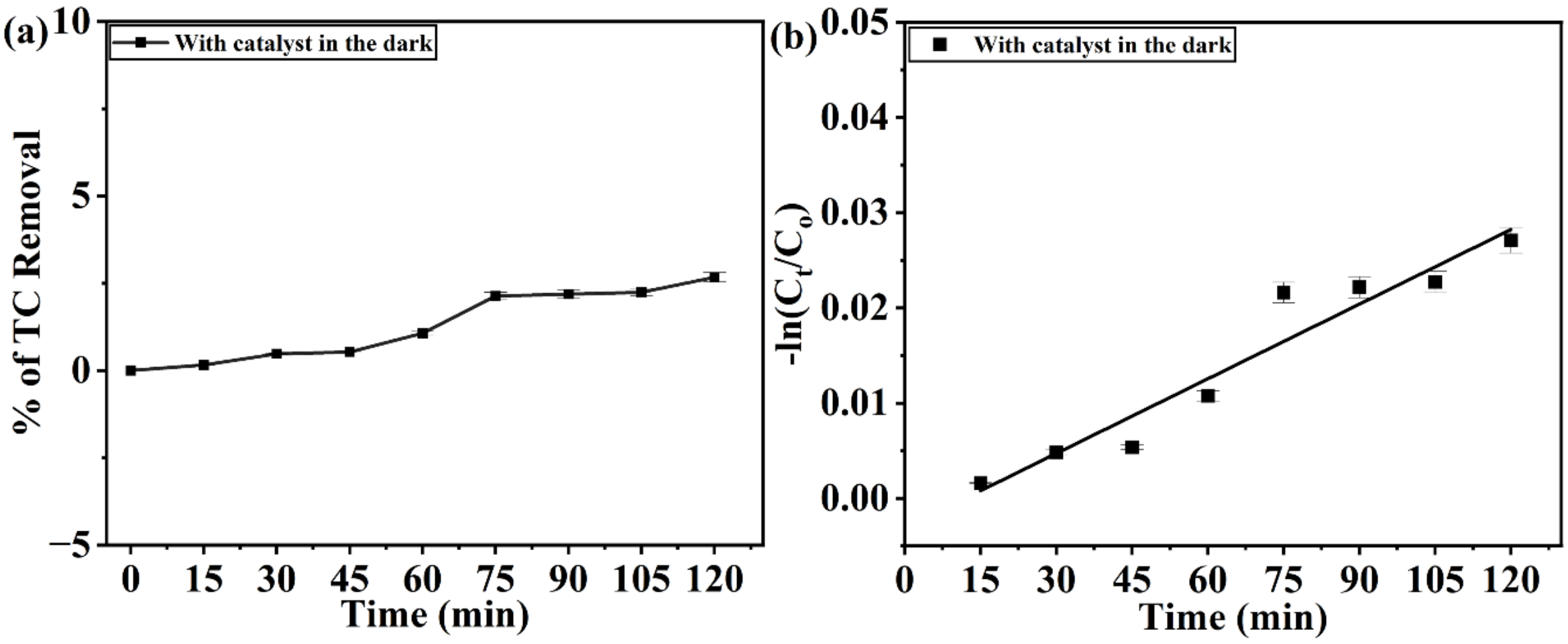

Fig. S2. (a) % of TC removal with time, (b) Pseudo-first-order kinetics model for TC removal of L-Histidine-MgO-AgI-5 in absence of sunlight; TC: 5 mg L^{-1} (100 mL), photocatalyst: 10 mg, pH: 7.

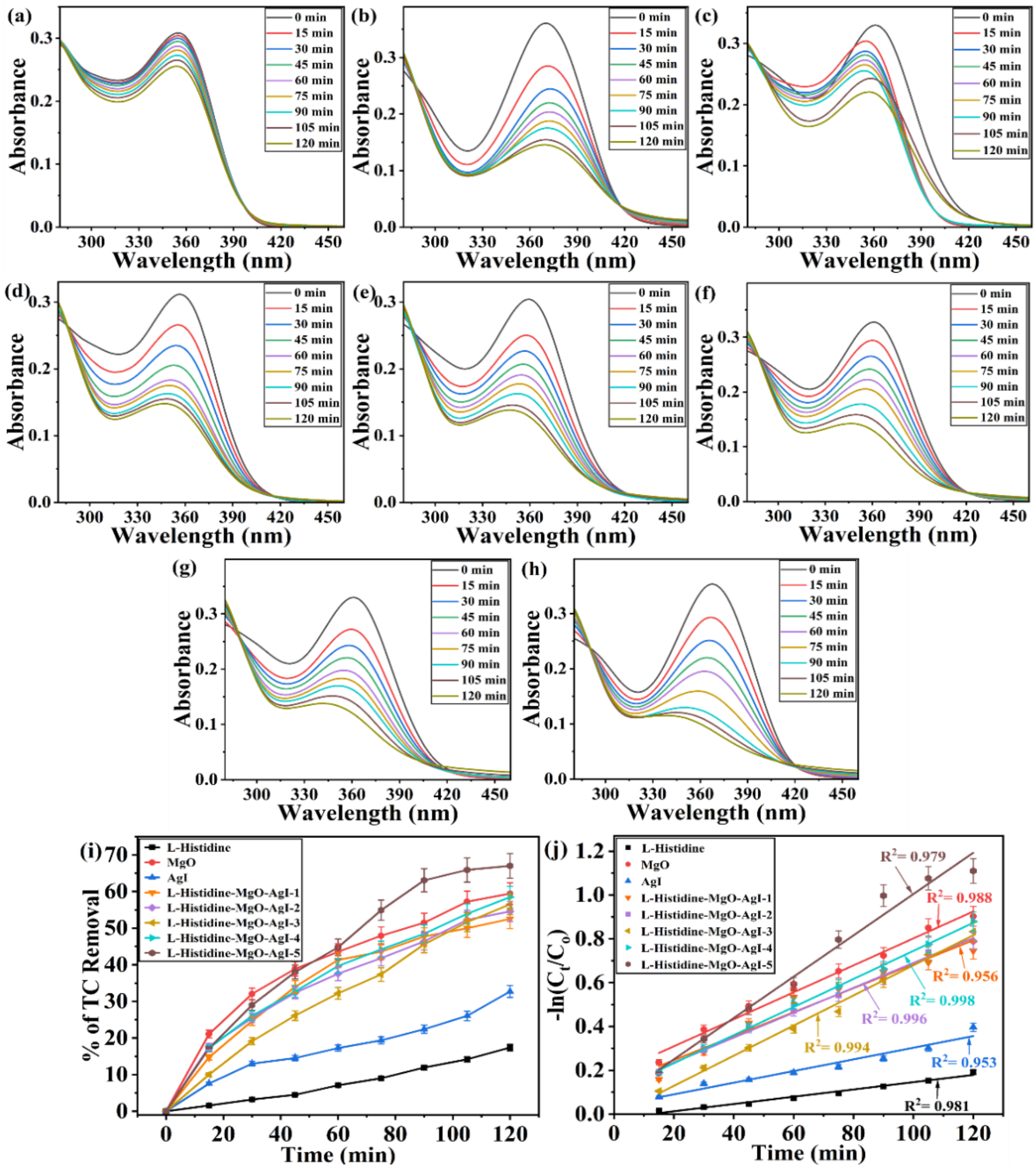

Fig. S3. Variations in the UV–vis absorption spectra of the TC solution under UV-lamp irradiation in the presence of (a) L-Histidine, (b) MgO, (c) AgI, (d) L-Histidine-MgO-AgI-1, (e) L-Histidine-MgO-AgI-2, (f) L-Histidine-MgO-AgI-3, (g) L-Histidine-MgO-AgI-4, and (h) L-Histidine-MgO-AgI-5, (i) % of TC removal with time, (j) Pseudo-first-order kinetics model for TC removal of L-Histidine, MgO, AgI, and all composite samples; TC: 5 mg L^{-1} (100 mL), photocatalyst: 10 mg, pH: 7.

Table S1 Pseudo-first-order rate constants (k) and correlation coefficients (R^2) for the photocatalytic degradation of TC by the individual components and the L-Histidine-MgO-AgI-x (x = 1-5) composites under sunlight and UV-lamp irradiation.

Samples	Sunlight irradiation		UV-lamp irradiation	
	k (min^{-1})	R^2	k (min^{-1})	R^2
L-Histidine	2.6×10^{-3}	0.987	1.66×10^{-3}	0.981
MgO	6.94×10^{-3}	0.995	6.15×10^{-3}	0.988
AgI	4.6×10^{-3}	0.942	2.66×10^{-3}	0.953
L-Histidine-MgO-AgI-1	5.37×10^{-3}	0.994	5.47×10^{-3}	0.956
L-Histidine-MgO-AgI-2	5.4×10^{-3}	0.993	5.68×10^{-3}	0.996
L-Histidine-MgO-AgI-3	6.48×10^{-3}	0.984	6.87×10^{-3}	0.994
L-Histidine-MgO-AgI-4	6.81×10^{-3}	0.996	6.41×10^{-3}	0.998
L-Histidine-MgO-AgI-5	11.11×10^{-3}	0.977	9.43×10^{-3}	0.979

Table S2 Pseudo-first-order rate constants (k) and correlation coefficients (R^2) for the photocatalytic degradation of TC by the L-Histidine-MgO-AgI-5 composite under varying operating parameters.

Experimental parameter	Condition	k (min^{-1})	R^2
Initial TC concentration	5 mg/L	11.11×10^{-3}	0.977
	10 mg/L	6.15×10^{-3}	0.982
	15 mg/L	6.42×10^{-3}	0.982
	20 mg/L	5.34×10^{-3}	0.934
	25 mg/L	5.56×10^{-3}	0.942
Catalyst dosage	5 mg	4.1×10^{-3}	0.997
	10 mg	5.56×10^{-3}	0.942
	15 mg	6.82×10^{-3}	0.967
pH	4	1.25×10^{-3}	0.986
	7	4.27×10^{-3}	0.999
	10	8.14×10^{-3}	0.996
Interfering ion	Non-ionic	5.34×10^{-3}	0.934
	Mg^{2+}	7.37×10^{-3}	0.994
	Na^{+}	7.96×10^{-3}	0.99
	Cl^{-}	5.35×10^{-3}	0.991
	NO_3^{-}	5.12×10^{-3}	0.964
Temperature	40 °C	3.32×10^{-3}	0.987
	50 °C	3.78×10^{-3}	0.959

	60 °C	4.61×10^{-3}	0.996
Scavenger	No scavenger	6.15×10^{-3}	0.982
	IPA	4.22×10^{-3}	0.942
	EDTA-2Na	3.45×10^{-3}	0.989
	BQ	2.69×10^{-3}	0.999

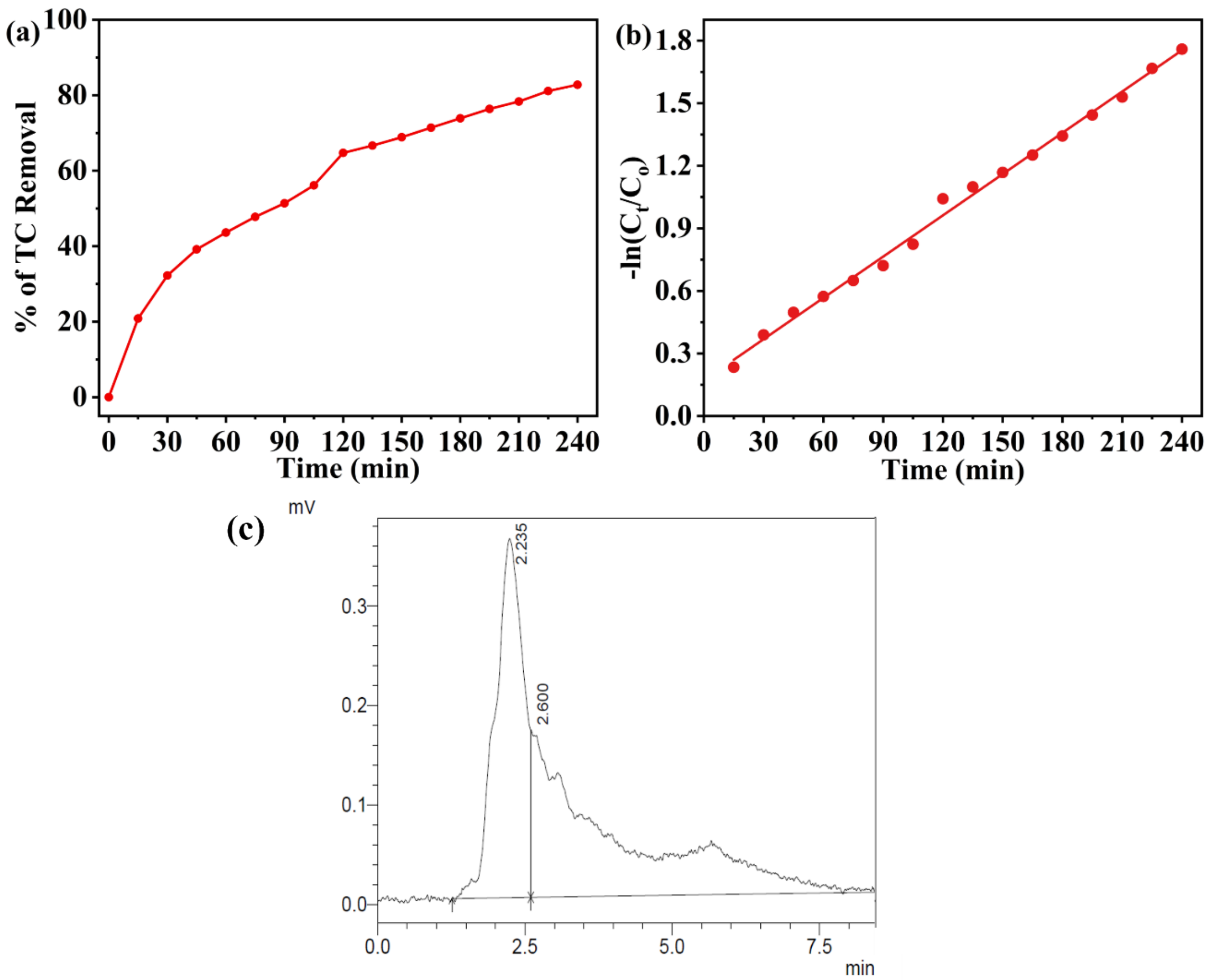

Fig. S4. (a) TC degradation efficiency versus irradiation time, (b) pseudo-first-order kinetic plot, and (c) HPLC chromatogram showing the retention time and peak area of TC used for quantification; TC: 10 mg L^{-1} (100 mL), photocatalyst (L-Histidine-MgO-AgI-5): 20 mg, pH: 7.

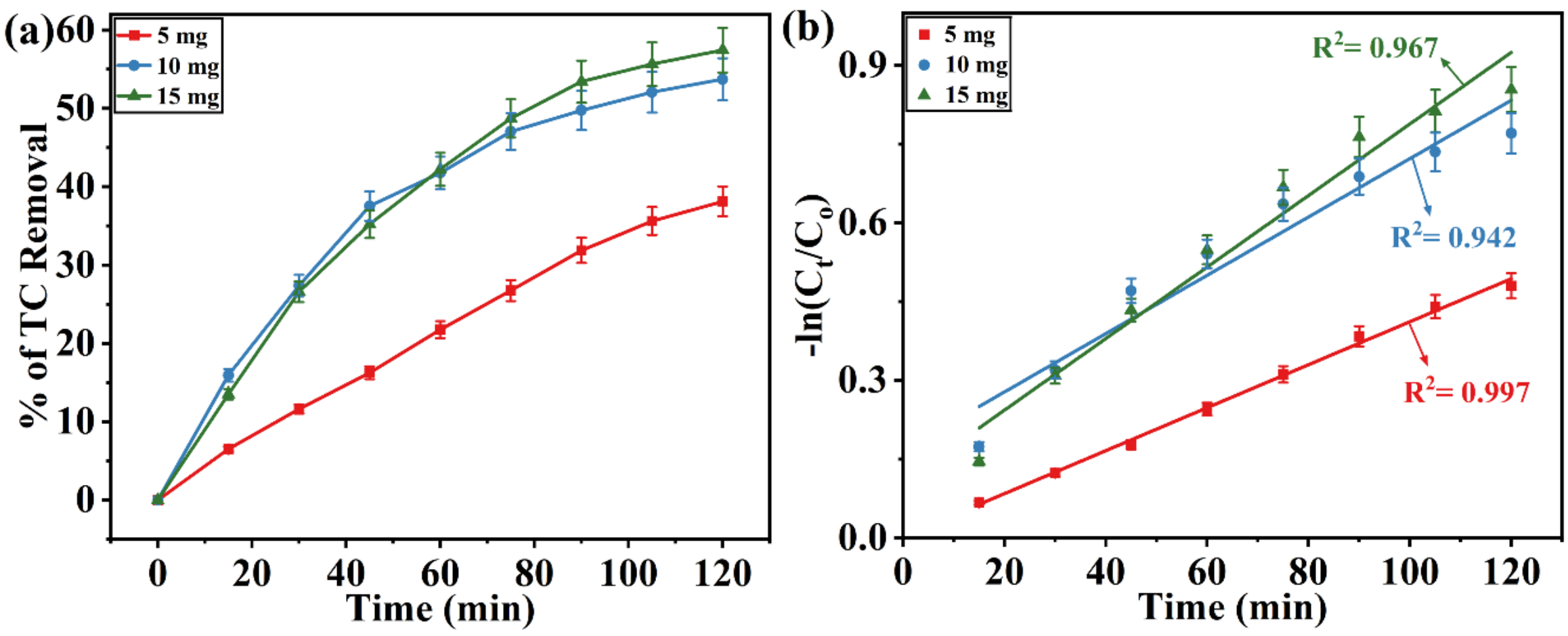

Fig. S5. (a) Influence of dosage on percentage of TC removal, (b) Pseudo-first-order kinetic model for TC removal at different dosages of L-Histidine-MgO-AgI-5; TC: 25 mg L^{-1} (100 mL), pH: 7.

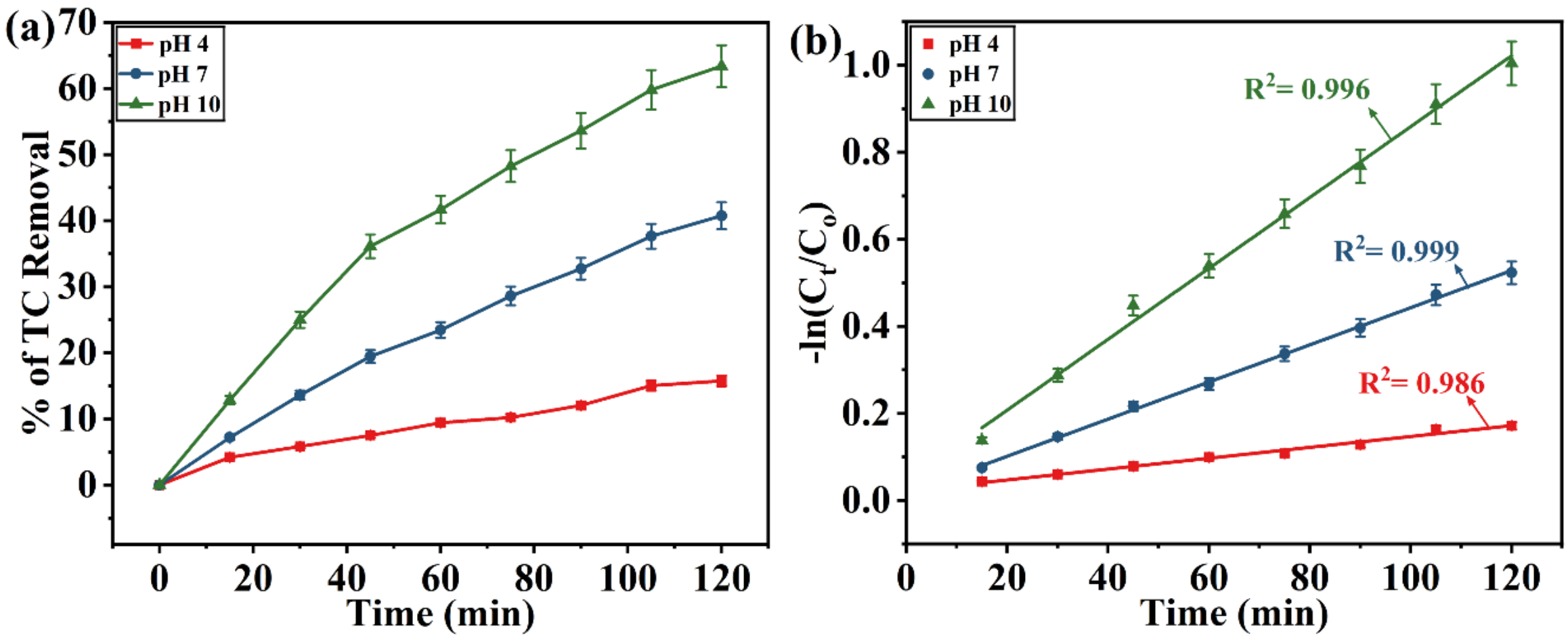

Fig. S6. (a) Effect of pH on percentage of TC removal, (b) Pseudo-first-order kinetic model for TC removal at three different pH (TC: 30 mg L^{-1} (100 mL), photocatalyst: 5 mg of L-Histidine-MgO-AgI-5).

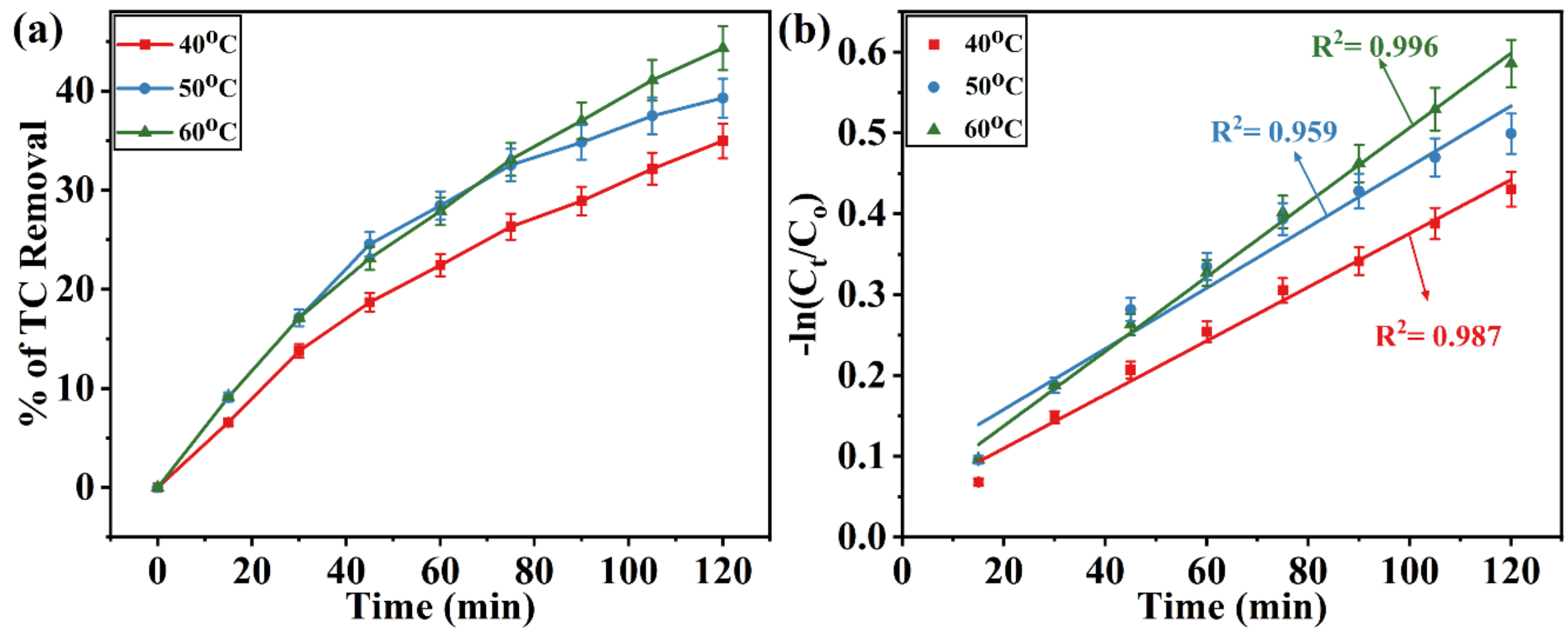

Fig. S7. (a) Effect of temperature on percentage of TC removal, (b) Pseudo-first-order kinetic model for TC removal at three different temperatures (TC: 30 mg L^{-1} (100 mL), photocatalyst: 10 mg of L-Histidine-MgO-AgI-5, pH: 7).

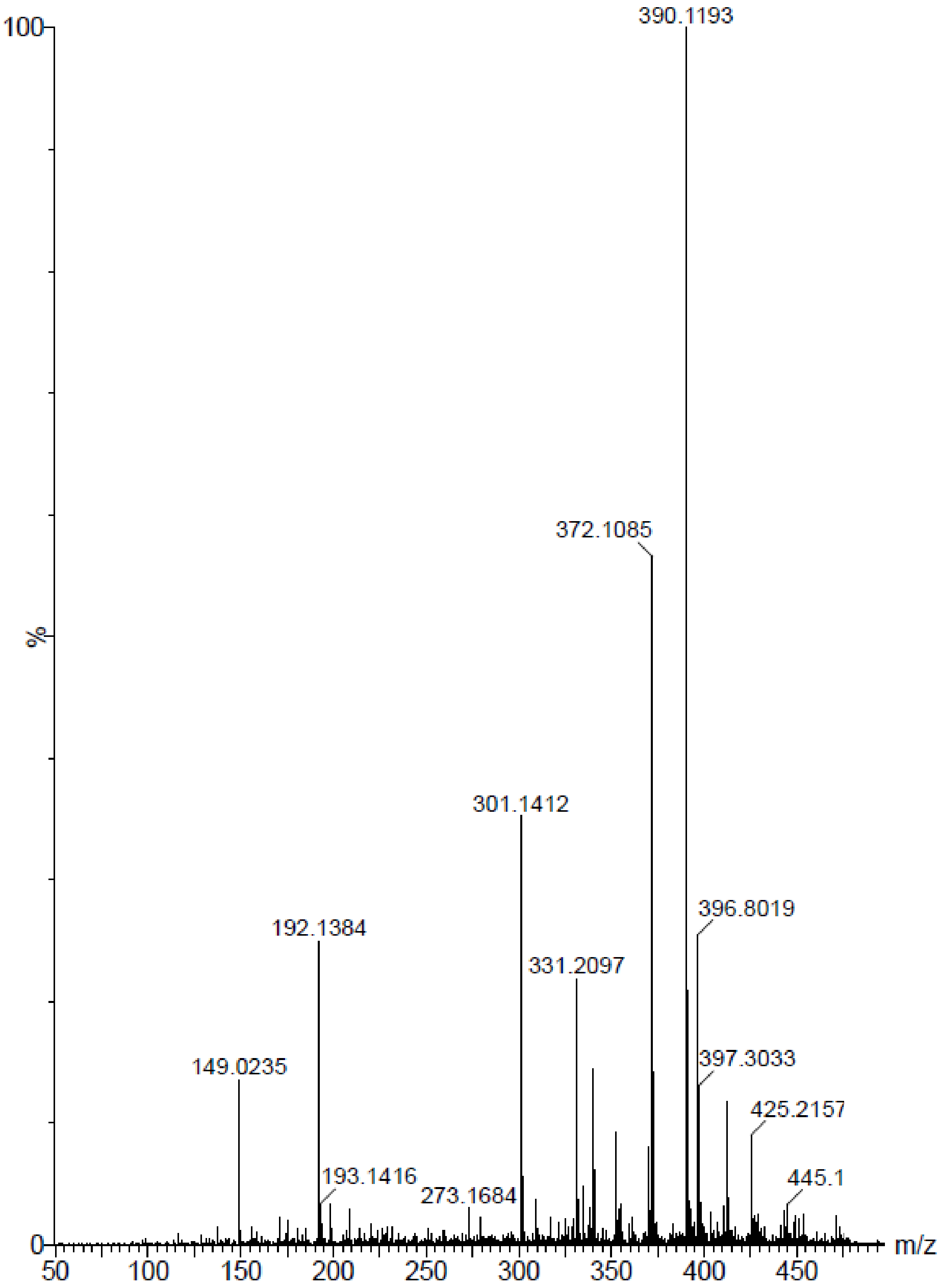

Fig. S8. The m/z values to realize the degradation intermediates using LC-MS.
